# Nanostructural diversity: self-assembly of isomeric pyrene–cholane amphiphiles into sheets, tubes, and worm-like morphologies†

**DOI:** 10.1039/d4ra06420h

**Published:** 2024-10-04

**Authors:** Edouard Ehret, Ioan Iacovache, Simon M. Langenegger, Benoît Zuber, Robert Häner

**Affiliations:** a Department of Chemistry, Biochemistry, Pharmaceutical Sciences, University of Bern Freiestrasse 3 CH-3012 Bern Switzerland robert.haener@unibe.ch https://www.haener.dcbp.unibe.ch/; b Institute for Anatomy, University of Bern Baltzerstrasse 2 CH-3012 Bern Switzerland

## Abstract

Phosphodiester-linked cholane–pyrene–cholane trimers self-assemble into sheet-, tube- and worm-like nanostructures in aqueous conditions. The nanotubes and worm-like assemblies exist as single- or multi-walled objects.

## Abstract 2

The supramolecular self-assembly of isomeric cholane–pyrene–cholane oligomers into different morphologies is described along as their spectroscopic properties.

## Graphical abstract

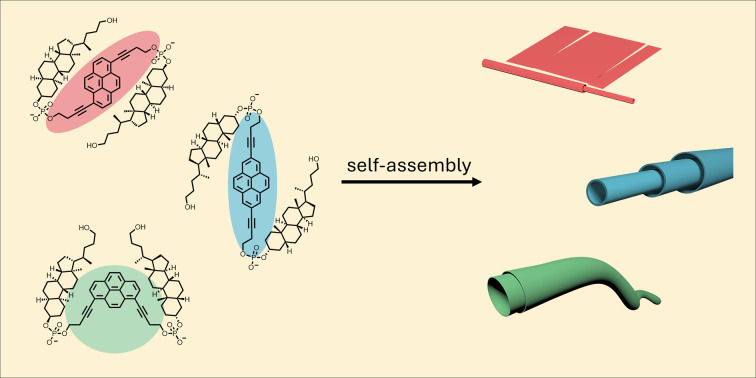

Supramolecular polymers (SP) have been extensively studied due to their unique properties and engineerability.^1–5^ They consist of recurring building blocks connected together through non-covalent interactions.^6,7^ Due to the nature of the interactions present upon self-assembly, they exhibit promising adaptable, reversible, self-healable, and recyclable characteristics.^8–12^ These attributes are of prime importance in the development of new optoelectronics and biomaterials. One class of building blocks consists of amphiphilic aromatic building blocks, which have been shown to self-assemble in aqueous conditions.^13–17^ Aromatic molecular units self-assemble in a wide variety of motifs, such as vesicles,^18–20^ fibres,^21–24^ sheets,^25,26^ ribbons,^27,28^ tubes,^29,30^ and toroids.^31,32^ Previously, our group has reported the self-assembly of supramolecular polymers from different oligoarenotide trimers.^33–37^ These systems are composed of three identical polyaromatic hydrocarbon moieties. In this work, we describe the self-assembly of phosphodiester-linked trimers in which two of the three polyaromatic hydrocarbon moieties were replaced by the non-aromatic, lipophilic building block cholane. The distinctive aspect of this trimer lies in the absence of possible intramolecular π–π interactions, which typically play a crucial role in the stabilization and organization of such systems. By eliminating these π–π interactions we aim to investigate the influence of alternative non-covalent interactions on the self-assembly process. The supramolecular self-assembly as well as the spectroscopic properties of three different cholane–pyrene–cholane trimers (Fig. 1) are presented herein.

**Fig. 1:**
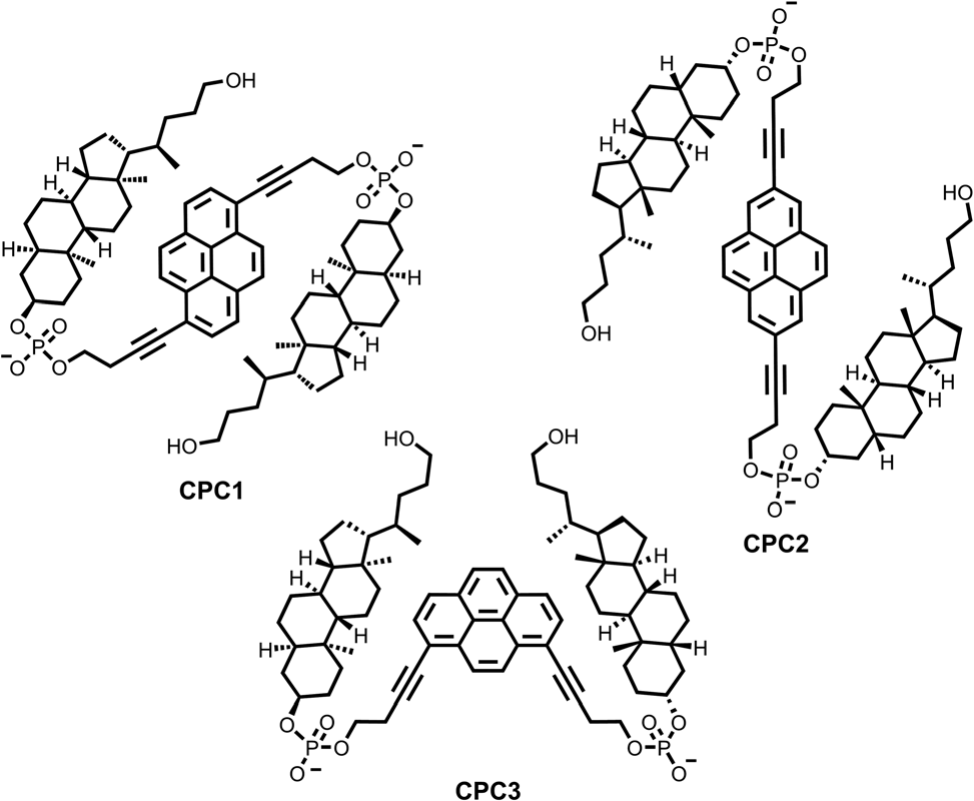
Chemical structures of the three synthesized cholane–pyrene–cholane trimers.

The three oligomers were synthesized according to published procedures^35,36^ purified by HPLC and analysed by HRMS (ESI).

The self-assembly process of all three trimers was studied by recording temperature dependent UV-vis absorption spectra (Fig. 2A). Upon cooling from 70 °C to 20 °C (0.5 °C min^−1^), characteristic changes are observed in the absorbance of the trimers. The appearance of a strong *J*-band is observed at 298 nm for CPC1 along with a hypochromicity of the bands at 355 nm to 405 nm at 20 °C. The spectroscopic changes associated with the aggregation of CPC1 are strongly reminiscent of previous observations made with 1,6-disubstituted pyrene trimers.^36^ Compounds CPC2 and CPC3, exhibit similar changes upon aggregation. Both derivatives exhibit a strong hypochromic effect. In addition, CPC2 displays a hypsochromic shift from 285 nm to 272 nm along with a bathochromic shift at 350 nm. For CPC3, a bathochromic shift of 3 to 6 nm is present for all absorption bands. Temperature-dependent excitation and emission fluorescence spectra are shown in Fig. 2B. Unlike previously reported for the 1,6-disubstituted pyrene trimers,^36^CPC1 does not exhibit any excimer and only very little monomer fluorescence upon self-assembly. In contrast, compounds CPC2 and CPC3 show fluorescence upon aggregation at 20 °C. In the case of CPC2, pyrene monomer emission is reduced by 50% upon self-assembly, whereas for CPC3, the pyrene monomer emission vanishes but the pyrene excimer emission (at 515 nm) is increased, indicating an interaction between the pyrene units upon aggregation. Minor excimer fluorescence observed at 70 °C can be ascribed to intermolecular interactions. The self-assembly processes of the different trimers were observed by monitoring the absorption changes at specific wavelength during the cooling process (Fig. S22, ESI†). The process follows the pattern of a nucleation–elongation process which starts at temperatures between 56 °C and 59 °C depending on the trimer.

To further analyse the supramolecular assemblies, atomic force microscopy (AFM) measurements were performed along with cryo-EM (Fig. 3). For the AFM measurements, the supramolecular polymers were deposited on a (3-aminopropyl)-triethoxysilane (APTES) modified mica sheets after temperature controlled self-assembly. The 1,6-disubstituted pyrene trimer (CPC1) results in the formation of tube- and sheet-like supramolecular polymers (Fig. 3A and S27, ESI†). The ratio of nanosheets and nanotubes, as well as their size, is influenced by the cooling rate applied during the sample preparation. A lower cooling rate (0.1 °C min^−1^), results in larger nanosheets and a higher fraction of them (see Fig. S28, ESI†) than observed after using a faster cooling rate (0.5 °C min^−1^). In both cases, the nanosheets are 2 nm high, while their side lengths vary between 0.5 to 5 μm. The height of 2 nm corresponds with the approximate molecular dimensions of the trimer (see Fig. S1, ESI†). The observed nanotubes are up to 2 μm long and possess a height up to 12 nm with increments of 4 nm. This last value correlates to a collapsed tube with a membrane thickness of 2 nm.^21^ In cryo-EM, only nanotubes can be observed (Fig. 3A and S31, ESI†). Moreover, they exist as single- and multi-walled species with diameters ranging between 30 and 80 nm. Sheets are not observed in cryo-EM images due to their small cross-section. CPC2 exhibits only one species in both, AFM and cryo-EM imaging (Fig. 3B, S29 and S32, ESI†) upon self-assembly. Similar to the assemblies formed by CPC1, CPC2 displays nanotubes with diameters ranging between 30 and 65 nm in cryo-EM. While in AFM measurements, the nanotubes are between 4 and 24 nm high, with increments of approximately 4 nm and up to 10 μm long. The membrane thickness of the tubes observed with CPC2, is the same as for CPC1, about 2 nm. Unlike its two other counterparts, the trimer containing the 1,8-disubstituted-pyrene (CPC3) forms nanostructures that are best described as worm-like, as observed by AFM and cryo-EM (Fig. 3C, S30 and S33, ESI†). These structures possess a height of 8 nm and a length of up to 20 μm. The worm-like nanostructures are composed of two membranes and have a diameter ranging between 70 and 180 nm. The SPs presumably collapse upon deposition on mica which results in the formation of four-layered objects with a total height of approximately 8 nm (see cross sections in Fig. 3C). The membrane thickness was therefore determined to be about 2 nm.

**Fig. 2:**
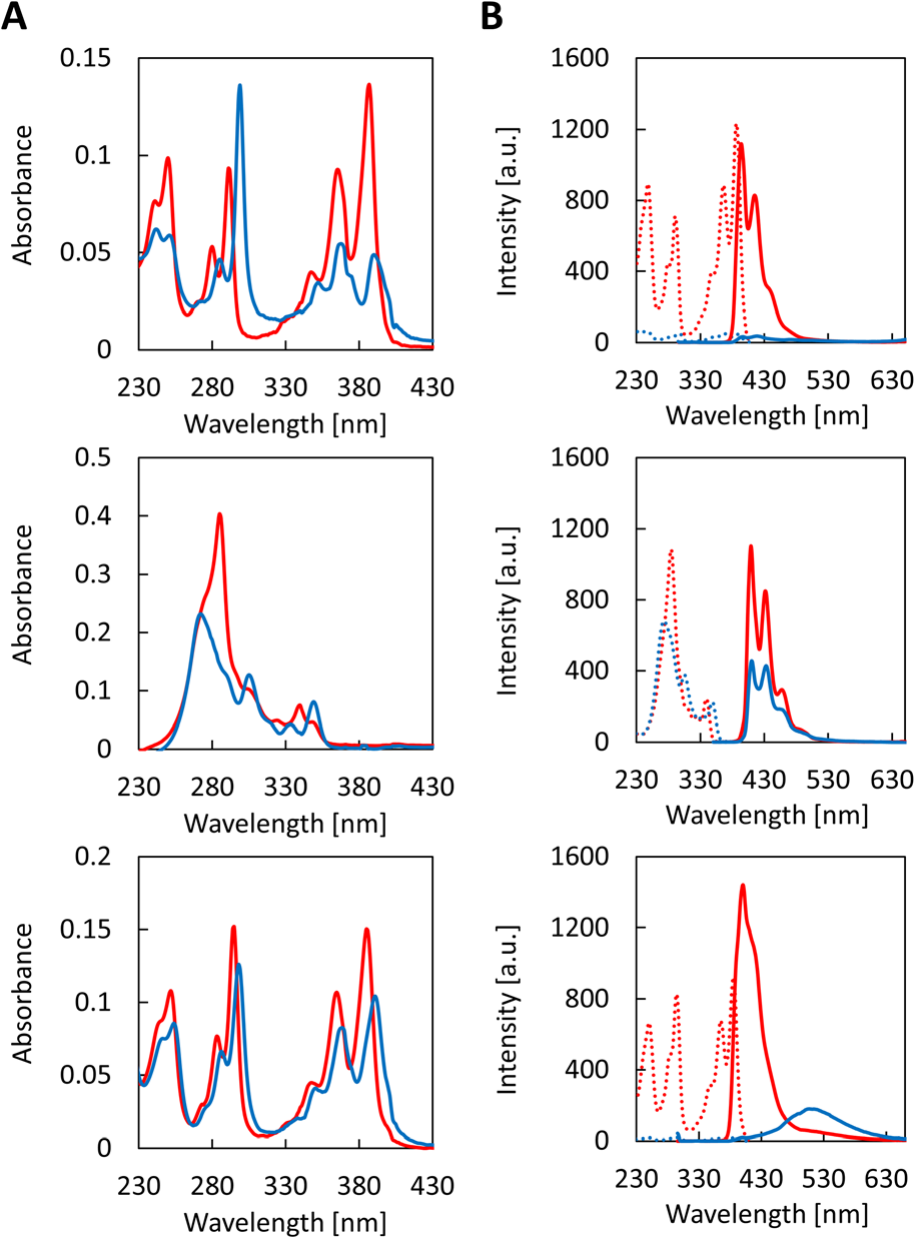
Temperature-dependant (A) UV-vis absorption spectra of the different oligomers at 70 °C (red) and 20 °C (blue) left: top CPC1, middle CPC2 and bottom CPC3; (B) fluorescence excitation spectra (dotted line) and fluorescence emission spectra (solid line) at 70 °C (red) and 20 °C (blue) right: top CPC1*λ*_ex_: 290 nm and *λ*_em_: 415 nm, middle CPC2*λ*_ex_: 284 nm and *λ*_em_: 409 nm and bottom CPC3*λ*_ex_: 294 nm and *λ*_em_: 415 nm. Conditions: 3 μM oligomer, 10 mM sodium phosphate buffer, pH 7.2, 10 mM NaCl and EtOH (20% for CPC1, 25% for CPC2 and 15% for CPC3).

**Fig. 3:**
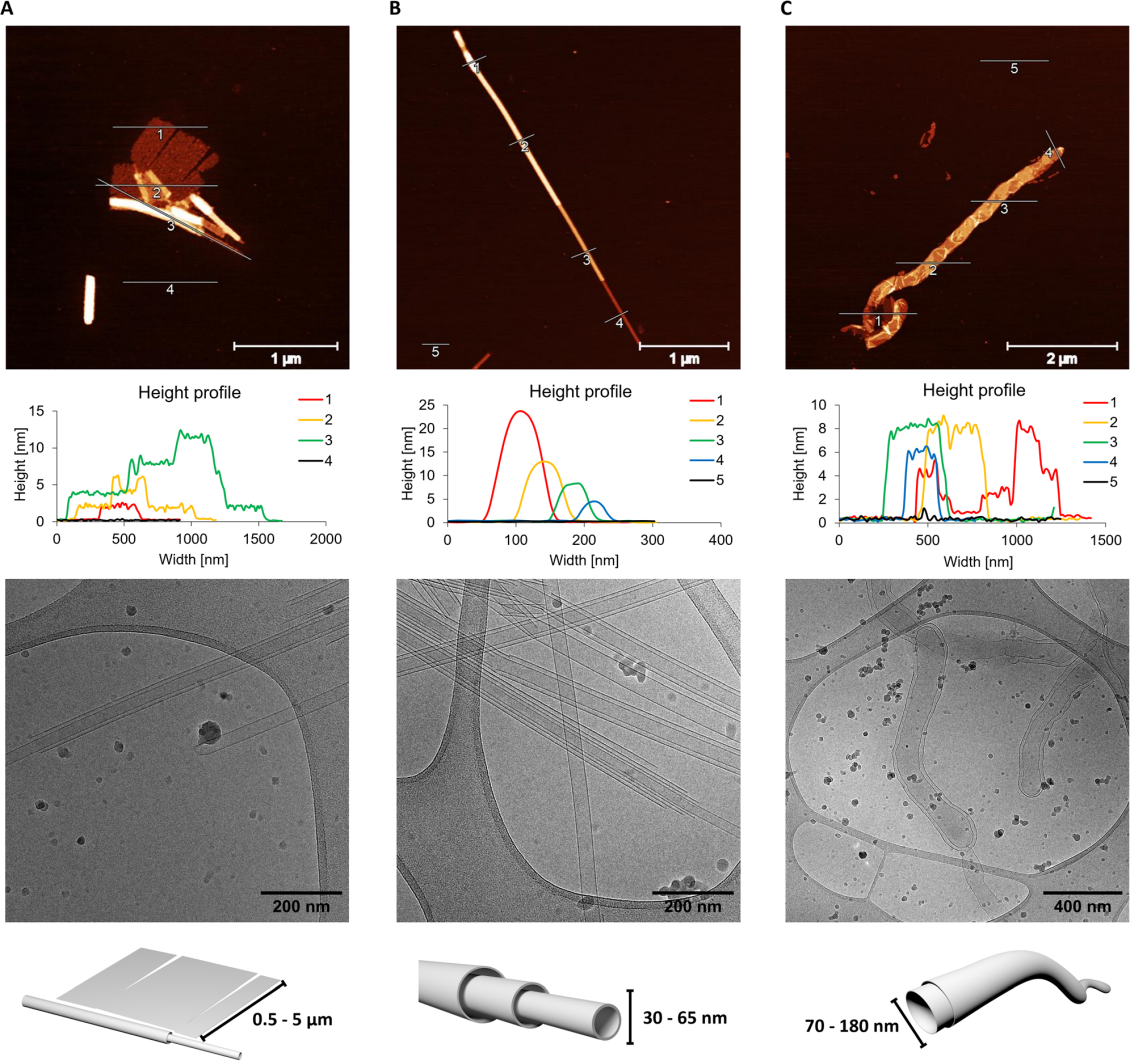
Microscopy measurements of oligomers CPC1 (A), CPC2 (B) and CPC3 (C). Atomic force microscopy (AFM) images with their corresponding cross sections (top), cryo-EM images (middle) and a schematic representation of the supramolecular polymer (bottom). Conditions: see Fig. 2; measurements were performed after thermal assembly (0.5 °C min^−1^).

In conclusion, the synthesis of three different phosphodiester-linked cholane–pyrene–cholane trimers and their self-assembly into various supramolecular objects have been described. The three different trimers assemble into nanosheets, nanotubes or worm-like structures depending on the pyrene isomer present in the trimer. A combination of sheets and tubes was observed with CPC1, the sheets possess a height of 2 nm and a width of up to 5 μm. Tubes are up to 80 nm wide and 2 μm long. For aggregates of CPC2, only nanotubes were observed under AFM and cryo-EM. They exist as single-and multi-walled (telescoped) nanotubes. They are between 30 and 65 nm wide and up to 10 μm long. Self-assemblies of the third trimer (CPC3) appear as worm-like nanostructures which possess two distinct membranes. The diameter varies between 70 to 180 nm and are up to 20 μm long. The results described herein show that short phosphodiester-based trimers of steroids and polyaromatic units self-assemble into a variety of supramolecular polymers in aqueous conditions. Furthermore, the usefulness of non-aromatic building blocks in the self-assembly of this type of amphiphilic trimers is demonstrated. Relatively little geometrical alterations of the substitution pattern of the aromatic pyrene moiety leads to significant morphological changes of the supramolecular polymers. Due to their well-defined structures and distinct spectroscopic properties, such supramolecular polymers bear the potential for applications in the areas of diagnostics, molecular electronics, or as artificial light-harvesting complexes.

## Data availability

The data supporting this article have been included as part of the ESI.†

## Conflicts of interest

There are no conflicts to declare.

## Supplementary Material

RA-014-D4RA06420H-s001
