# Supplementary material for: Nanostructural diversity: self-assembly of isomeric pyrene–cholane amphiphiles into sheets, tubes, and worm-like morphologies
Source: RSC Adv. 2024 Oct 4;14(43):31498–501. doi: 10.1039/d4ra06420h (PMC11450553; doi:10.1039/d4ra06420h)
Supplement: RA-014-D4RA06420H-s001 [file RA-014-D4RA06420H-s001.pdf]

Supplementary Information (SI) for RSC Advances.
This journal is © The Royal Society of Chemistry 2024

Electronic Supplementary Information

Nanostructural Diversity: Self-Assembly of Isomeric Pyrene-Cholane Amphiphiles into Sheets, Tubes, and Worm-like Morphologies.

Edouard Ehret,[a] Ioan Iacovache,[b] Simon M. Langenegger,[a] Benoît Zuber,[b] and Robert Häner[a]*

[a] Department of Chemistry, Biochemistry, and Pharmaceutical Sciences, University of Bern, Freiestrasse 3, CH-3012 Bern, Switzerland

[b] Institute of Anatomy, University of Bern, Baltzerstrasse 2, CH – 3012 Bern, Switzerland

Table of contents

1. General Methods 3
2. Synthesis of the Oligomers 4
  2.1. Organic Synthesis 4
  2.2. NMR Spectra 10
  2.3. MS Spectra 14
  2.4. Analytical HPLC Trace 17
3. Preparation of the sample 18
4. Temperature-dependent UV-vis Spectra 18
5. Atomic Force Microscopy 18
6. Cryo-EM 21
7. References 23

1. General Methods

All reagents and solvents were purchased from commercial sources and used without further purification. Water was used from a Milli-Q system. Mass spectra were measured by the Analytical Research and Services (ARS) of the University of Bern, Switzerland, on a Thermo Fisher LTQ Orbitrap XL using Nano Electrospray Ionization (NSI). Mass spectra were measured in positive and negative ion mode in mixtures of acetonitrile/water/triethylamine. UV-Vis spectra were measured on a Jasco V-730 spectrophotometer using quartz cuvettes with an optical path of 1 cm. Fluorescence spectra were collected on a Jasco spectrophotometer FP-8300 using an excitation slit of 1 nm and emission slit of 2.5 nm. Supramolecular self-assembly was carried-out via thermal disassembly and reassembly. The sample solution was heated to 70 °C, then cooled with a gradient of 0.5 °C/min to 20 °C (unless indicated differently) in a thermostat equipped with a Peltier. Atomic force microscopy (AFM) experiments were conducted under ambient conditions on a Nanosurf FlexAFM instrument using tapping mode. AFM samples were prepared on (3-aminopropyl)triethoxysilane (APTES)-modified mica sheets (Glimmer "V1", 20 mm x 20 mm, G250-7, Plano GmbH) according to published procedures.[1] The mica sheets were freshly cleaved and mounted with tape on top of a desiccator (3 L), before the desiccator was purged with argon. APTES (30 μL) was pipetted into an Eppendorf tube cap and Hünig's base (10 μL) was added into a second cap. Both Eppendorf tube caps were placed at the bottom of the desiccator below the mica sheets, then the desiccator was closed. The mica sheets were left for one night in the desiccator to cure. Afterwards, the corresponding sample solution (20 μL) was pipetted onto the APTES-modified mica sheet. After an adsorption time of 10 min, the mica sheet was rinsed with Milli-Q water (2 mL), then dried under a stream of argon. Samples for cryo-EM were plunge frozen using the FEI Vitrobot Mark 4 at room temperature and 100% humidity. In brief, copper lacey carbon grids were glow discharged (air -10 mA for 20 seconds). 3 μL of the sample were pipetted on the grids and blotted for 3 seconds before plunging into liquid ethane. Sample grids were stored in liquid nitrogen. Images were acquired using a Gatan 626 cryo holder on a Falcon III equipped FEI Tecnai F20 in nanoprobe mode. Due to the nature of the sample, acquisition settings had to be adjusted for a low total electron dose (less than 20 $e^-/Å^2$) using EPU software. Distance measurements were done with Fiji[2,3] using the multi-point tool to set marks. The HPLC traces and the mass spectra of **CPC1-CPC3** are displayed in Figure S18-Figure S21. The ethanol content used in the buffer medium was adjusted for each trimer individually to ensure an experimentally workable temperature window (i.e. completely de-aggregated form at 70 °C and fully aggregated form at 20 °C).

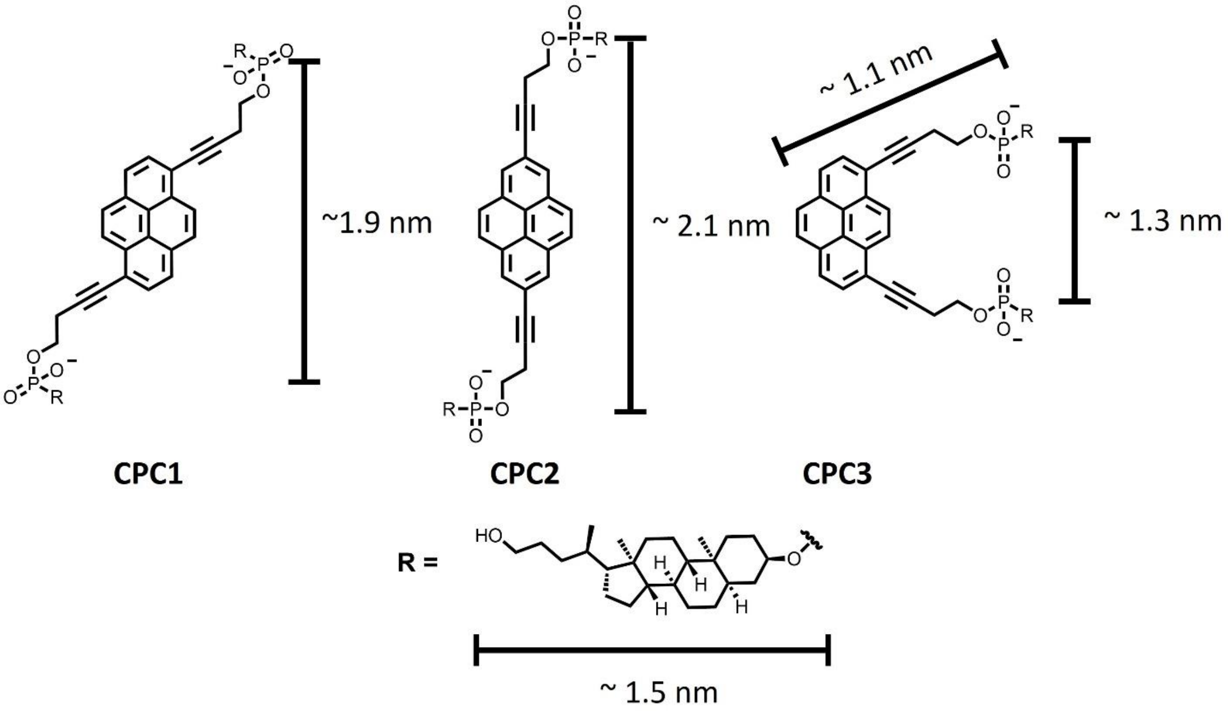

Figure S1: Length of the different molecules (determined with Chem3D).

2. Synthesis of the Oligomers

After their deprotection, the different oligomers were purified by reverse-phase HPLC (Shimadzu LC-20AT, ReproSil 100 C8, 5,0 µm, 250×4 mm) at 40 °C with a flow rate of 1 mL/min. Solvent A: aqueous 2.1 mM triethylamine (TEA) / 25 mM 1,1,1,3,3,3-hexafluoropropan-2-ol (HFIP) pH 8; solvent B: acetonitrile; gradient: B [%] t_R [min]: 35 (0), 35 (2), 65 (24). The purified cholane-pyrene-cholane trimers **CPC1-CPC3** were dissolved in 1 mL of 80% EtOH in Milli-Q H_2O. Afterwards, the absorbance of the conjugates was measured to determine the concentration of the stock solutions **CPC1-CPC3**. The Beer-Lambert law was applied to determine the concentrations with the molar absorption coefficients in $L \cdot mol^{-1} \cdot cm^{-1}$ were used: ε(1,6-Py, 384 nm): 54’000; ε(2,7-Py, 260 nm): 32’000; ε(1,8-Py, 383 nm): 62’000.

2.1. Organic Synthesis

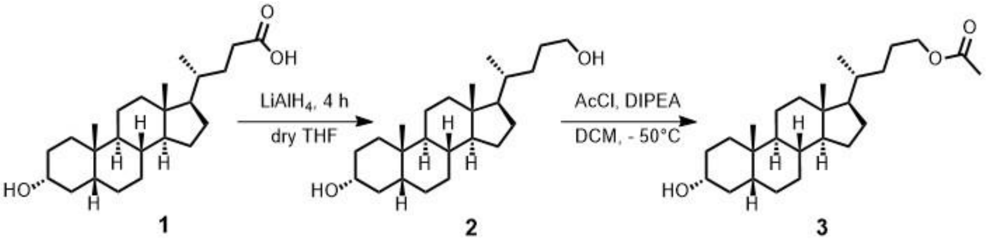

Figure S2 Synthesis pathway for compound **3**.

(3R,8R,9S,10S,13R,14S,17R)-17-((R)-5-hydroxypentan-2-yl)-10,13-dimethylhexadecahydro-1H-cyclopenta[a]phenanthren-3-ol (**2**) Synthesized according to published literature.[4]

$LiAlH_4$ (474 mg, 12.5 mmol, 4.7 eq.) was added slowly to a solution of lithocholic acid, **1** (1 g, 2.7 mmol, 1 eq.) in anhydrous THF (60 mL) at 0 °C. The reaction mixture was then stirred for 2.5 h at RT. H_2O (0.5 mL),

15 % aqueous NaOH (0.5 mL), and more H_2O (1.5 mL) were added sequentially to the reaction mixture. The white solid was removed by filtration. The organic layer was dried over Na_2SO_4, filtered, and concentrated under reduced pressure. The white solid was not further purified and was used as such in the next step (693 mg, 1.91 mmol, 72%). ^1H NMR **2** (300 MHz, $CDCl_3$) δ = 3.70 – 3.52 (m, 3H), 2.02 – 0.93 (m, 24H), 0.94 – 0.89 (m, 6H), 0.64 (s, 3H).

(4R)-4-((3R,8R,9S,10S,13R,14S,17R)-3-hydroxy-10,13-dimethylhexadecahydro-1H-cyclopenta[a]phenanthren-17-yl)pentyl acetate (**3**)

Acetyl chloride (23 µL, 0.33 mmol 1.2 eq.) dissolved in anhydrous THF (0.5 mL) was added dropwise under Ar to a mixture at -50 °C (dry ice, 1:1 ethanol/acetone) of **2** (99 mg, 0.27 mmol, 1 eq.) and DIPEA (0.1 mL, 0.59 mmol, 2.1 eq.) in anhydrous THF (4.5 mL). The reaction was stirred for 3 h at -50 °C, followed by 1 h at RT. The reaction mixture was then added to 2 mL of HCl (3.7%) and extracted with diethylether (3 x 10 mL). The organic phase was dried over Na_2SO_4, filtered, and concentrated under reduced pressure. The crude was purified by column chromatography (SiO_2, heptane/EtOAc 8:2; Rf=0.16). Compound **3** was isolated as a white solid (50 mg, 45%). ^1H NMR **3** (300 MHz, $CDCl_3$) δ = 4.01 (m, 2H), 3.62 (m, 1H), 2.04 (s, 3H), 2.00 – 0.94 (m, 21H), 0.91 (m, 6H), 0.64 (s, 3H). HRMS-NSI (*m/z*): $[M+K]^+$ calcd for $C_{26}H_{44}O_3K$, 443.2922; found, 433.2929.

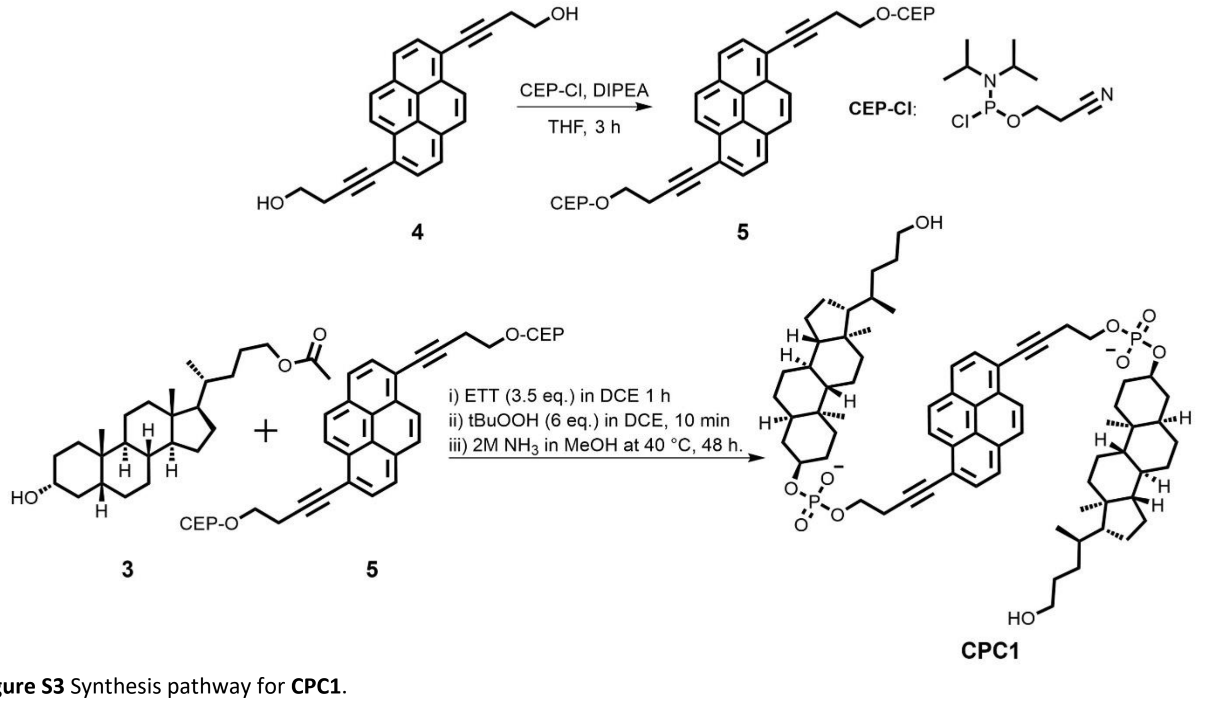

Figure S3 Synthesis pathway for **CPC1**.

Bis(2-cyanoethyl)(pyrene-1,6-diylbis(but-3-yne-4,1-diyl))bis(diisopropylphosphoramidite) (**5**)

Compound **4** (50 mg, 0.15 mmol, 1 eq.) was dissolved in anhydrous THF (3 mL) and DIPEA (0.3 mL, 1.48 mmol, 10 eq.). 2-Cyanoethyl N,N-diisopropylchlorophosphoramidite (CEP-Cl, 72.5 µL, 0.33 mmol, 2.2 eq.) was added dropwise at RT and the reaction mixture stirred for 3 h under argon. The reaction mixture was concentrated under reduced pressure. The resultant yellow-greenish foam was purified by a short flash column chromatography (SiO_2, heptane/EtOAc 7:3 + 1% NEt_3). Compound **5** was isolated as a yellow oil (89 mg, 81%). ^{1}H NMR **5** (300 MHz, $CDCl_3$) δ =8.60 (d, J = 9.1 Hz, 2H), 8.18 – 8.06 (m, 6H), 4.13 – 3.81 (m, 9H), 3.70 (m, 4H), 3.00 (t, J = 6.8 Hz, 4H), 2.67 (m, 5H), 1.32 – 1.20 (m, 33H). ^{31}P NMR **5** (121 MHz, $CDCl_3$) δ = 148.25. HRMS-NSI (*m/z*): $[M+H]^+$ calcd for $C_{42}H_{53}N_4O_4P_2$, 739.3537; found, 739.3553.

Oligomer **CPC1**

To a solution of **3** (33 mg, 0.08 mmol, 3 eq.) and **5** (20 mg, 0.03 mmol, 1 eq.) in 1,2 dichloroethane (DCE, 0.83 mL) was added a solution of 5-(ethylthio)-1H-tetrazole (12 mg, 0.09 mmol, 3.5 eq.) in DCE (0.31 mL) under argon atmosphere. The reaction was stirred at RT for 1 h after which a solution of ^{t}BuOOH (70% in water, 23 µL, 0.16 mmol) was added. After 10 minutes, the reaction mixture was diluted with $CHCl_3$ (15 mL) and washed with aq. sat. $NaHCO_3$ (15 mL) and brine (15 mL). The organic layer was dried over $MgSO_4$, filtrated, and concentrated under reduced pressure. The crude was purified by preparative TLC (DCM/toluene/MeOH 86:10:4). The protected oligomer was isolated as a brown solid. It was further deprotected with 2 M NH_3 in MeOH (6 mL) for 48 h at 40 °C and purified by RP-HPLC. HRMS-NSI (*m/z*): $[M-H]^-$ calcd for $C_{72}H_{98}O_{10}P_2$, 592.3323; found, 592.3315.

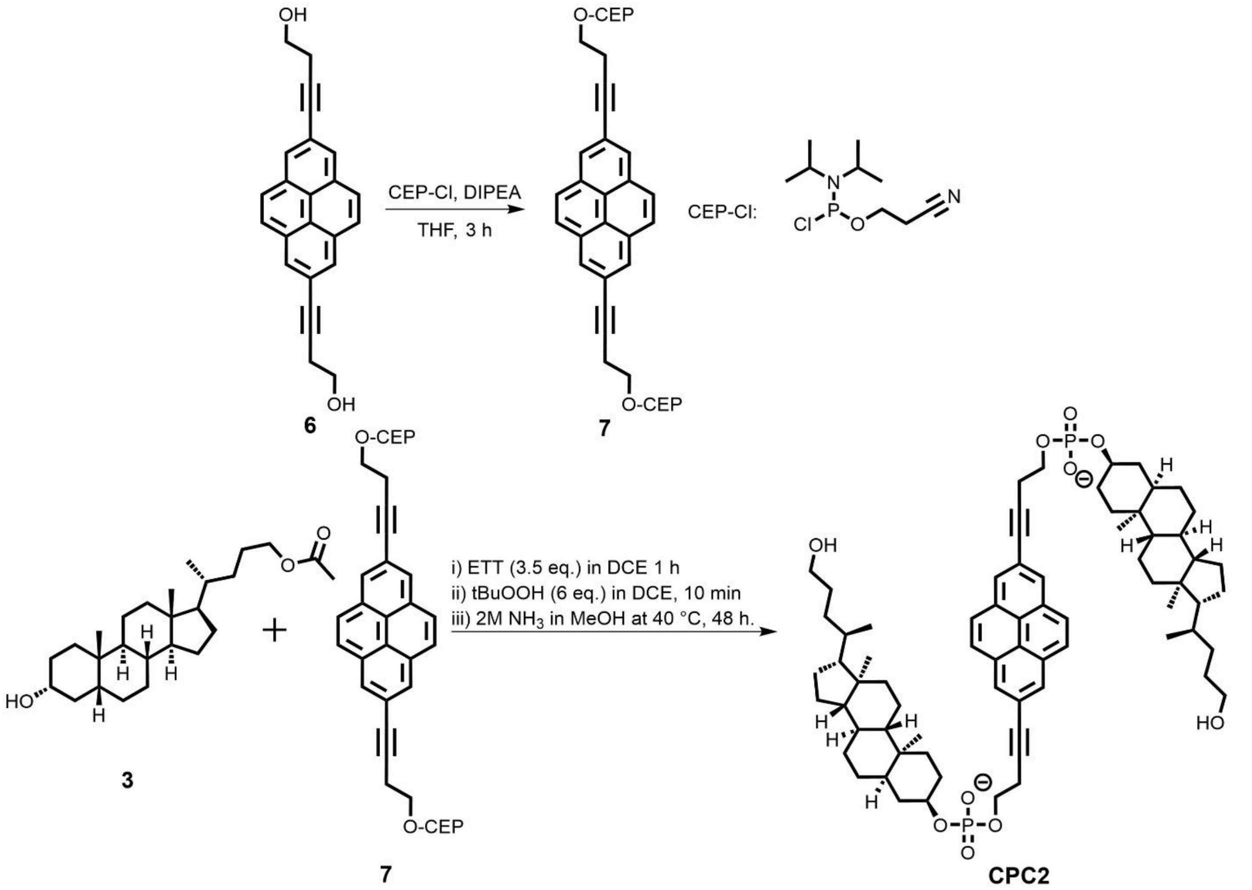

Figure S4 Synthesis pathway for **CPC2**.

Bis(2-cyanoethyl) (pyrene-2,7-diylbis(but-3-yne-4,1-diyl)) bis(diisopropylphosphoramidite) (**7**)

Compound **6** (100.3 mg, 0.27 mmol, 1 eq.) was dissolved in anhydrous THF (6 mL) and DIPEA (0.5 mL, 2.93 mmol, 10 eq.). 2-Cyanoethyl N,N-diisopropylchlorophosphoramidite (CEP-Cl, 145.5 µL, 0.65 mmol, 2.2 eq.) was added dropwise at RT and the reaction mixture stirred for 3 h under argon. The reaction mixture was concentrated under reduced pressure. The resultant yellow-greenish foam was purified by a short flash column chromatography (SiO_2, heptane/EtOAc 7:3 + 1% NEt_3). Compound **7** was isolated as a yellow oil (190 mg, 87%). ^{1}H NMR **7** (300 MHz, $CDCl_3$) δ = 8.18 (s, 4H), 7.98 (s, 4H), 4.05 – 3.78 (m, 6H), 3.67 (m, 3H), 2.85 (t, J=6.9, 4H), 2.67 (m, 4H), 1.23 (dd, J=6.8, 5.4, 27H). ^{31}P NMR **7** (121 MHz, $CDCl_3$) δ = 148.23. HRMS-NSI (*m/z*): $[M+H]^+$ calcd for $C_{42}H_{53}N_4O_4P_2$, 739.3537; found, 739.3546.

Oligomer **CPC2**

To a solution of **3** (49 mg, 0.12 mmol, 3 eq.) and **7** (30 mg, 0.04 mmol, 1 eq.) in DCE (1.24 mL) was added a solution of 5-(ethylthio)-1H-tetrazole (18.5 mg, 0.14 mmol, 3.5 eq.) in DCE (0.47 mL) under argon atmosphere. The reaction was stirred at RT for 1 h after which a solution of ^{t}BuOOH (70% in water, 34 µL, 0.24 mmol) was added. After 10 minutes, the reaction mixture was diluted with $CHCl_3$ (15 mL) and washed with aq. sat. $NaHCO_3$ (15 mL) and brine (15 mL). The organic layer was dried over $MgSO_4$, filtrated, and concentrated under reduced pressure. The crude was purified by preparative TLC (DCM/toluene/MeOH 88:10:2). The protected oligomer was isolated as a brown solid. It was further deprotected with 2 M NH_3

in MeOH (6 mL) for 48 h at 40 °C and purified by RP-HPLC. HRMS-NSI (*m/z*): $[M-H]^-$ calcd for $C_{72}H_{98}O_{10}P_2$, 592.3323; found, 592.3312.

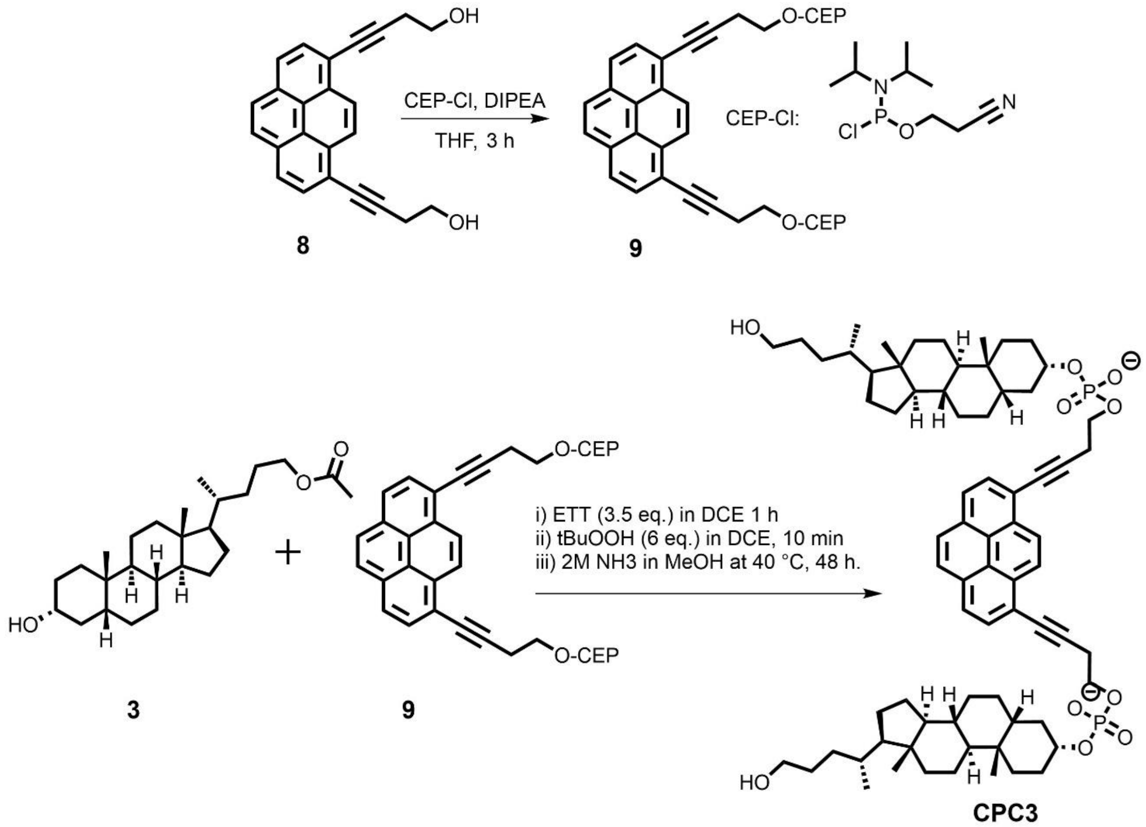

Figure S5 Synthesis pathway for **CPC3**.

Bis(2-cyanoethyl)(pyrene-1,8-diylbis(but-3-yne-4,1-diyl))bis(diisopropylphosphoramidite) (**9**)

Compound **8** (20 mg, 0.06 mmol, 1 eq.) was dissolved in anhydrous THF (2 mL) and DIPEA (0.2 mL, 0.59 mmol, 10 eq.). 2-Cyanoethyl N,N-diisopropylchlorophosphoramidite (CEP-Cl, 29 µL, 0.13 mmol, 2.2 eq.) was added dropwise at RT and the reaction mixture stirred for 3 h under argon. The reaction mixture was concentrated under reduced pressure. The resultant yellow-greenish foam was purified by a short flash column chromatography (SiO_2, heptane/EtOAc 7:3 + 1% NEt_3). Compound **9** was isolated as a yellow oil (39 mg, 89%). ^1H NMR **9** (300 MHz, $CDCl_3$) δ = 8.60 (d, J=9.1, 2H), 8.18 – 8.06 (m, 6H), 4.13 – 3.81 (m, 8H), 3.70 (m, 4H), 3.00 (t, J=6.8, 4H), 2.67 (m, 5H), 1.32 – 1.20 (m, 33H). ^{31}P NMR **9** (121 MHz, $CDCl_3$) δ = 148.25. HRMS-NSI (*m/z*): $[M+H]^+$ calcd for $C_{42}H_{53}N_4O_4P_2$, 739.3537; found, 739.3550.

Oligomer **CPC3**

To a solution of **3** (33 mg, 0.08 mmol, 3 eq.) and **9** (20 mg, 0.03 mmol, 1 eq.) in DCE (0.83 mL) was added a solution of 5-(ethylthio)-1H-tetrazole (12 mg, 0.09 mmol, 3.5 eq.) in DCE (0.31 mL) under argon atmosphere. The reaction was stirred at RT for 1 h after which a solution of ^tBuOOH (70% in water, 23 µL, 0.16 mmol) was added. After 10 minutes, the reaction mixture was diluted with $CHCl_3$ (15 mL) and washed with aq. sat. $NaHCO_3$ (15 mL) and brine (15 mL). The organic layer was dried over $MgSO_4$, filtrated, and

concentrated under reduced pressure. The crude was purified by preparative TLC (DCM/toluene/MeOH 86:10:4). The protected oligomer was isolated as a brown solid. It latter was further deprotected with 2 M NH_3 in MeOH (6 mL) for 48 h at 40 °C and purified by RP-HPLC. HRMS-NSI (*m/z*): $[M-H]^-$ calcd for $C_{72}H_{98}O_{10}P_2$, 592.3323; found, 592.3312.

2.2. NMR Spectra

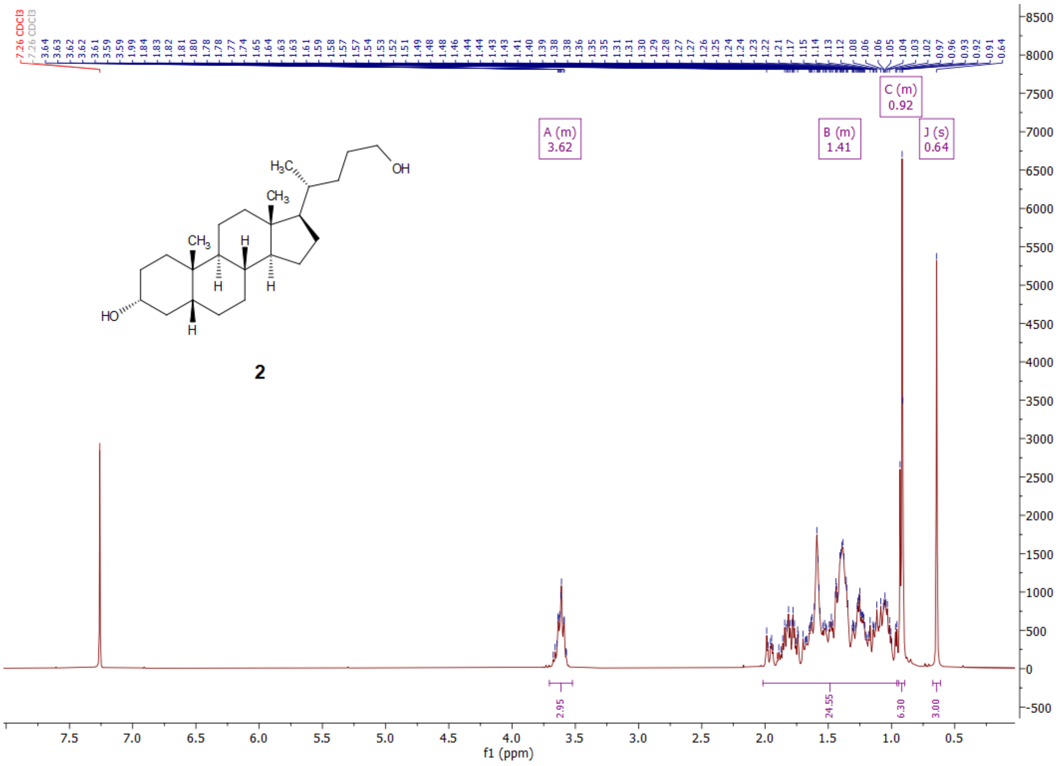

Figure S6 ^{1}H NMR spectra of **2**.

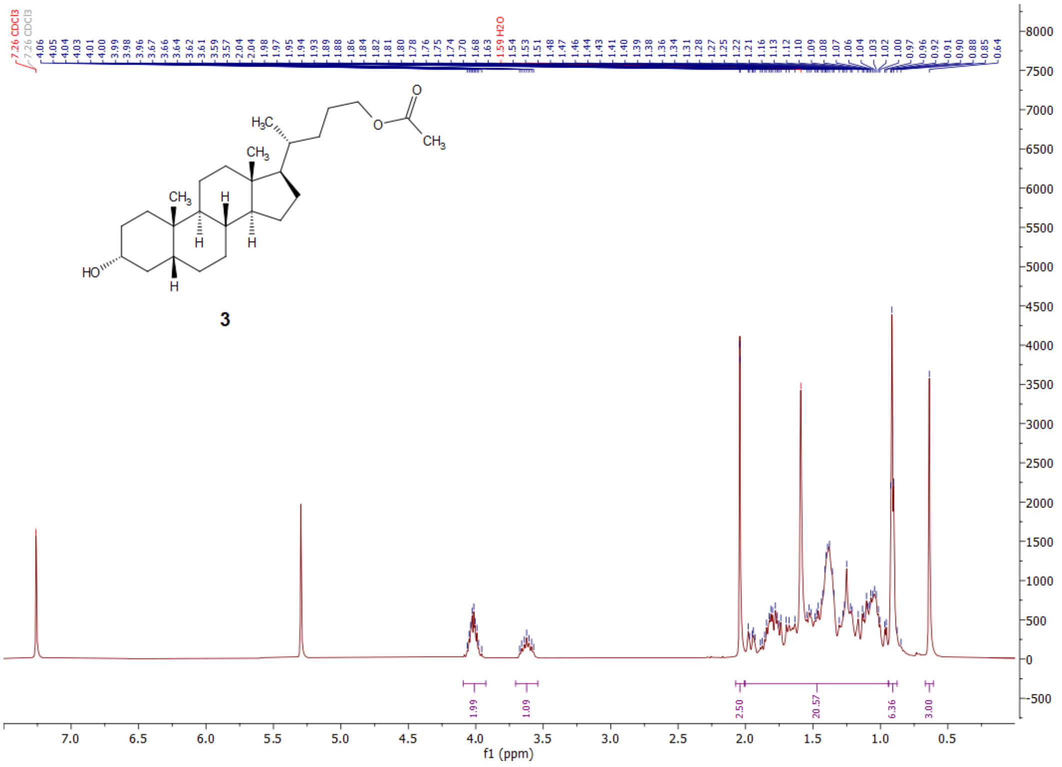

Figure S7 ^{1}H NMR spectra of **3**.

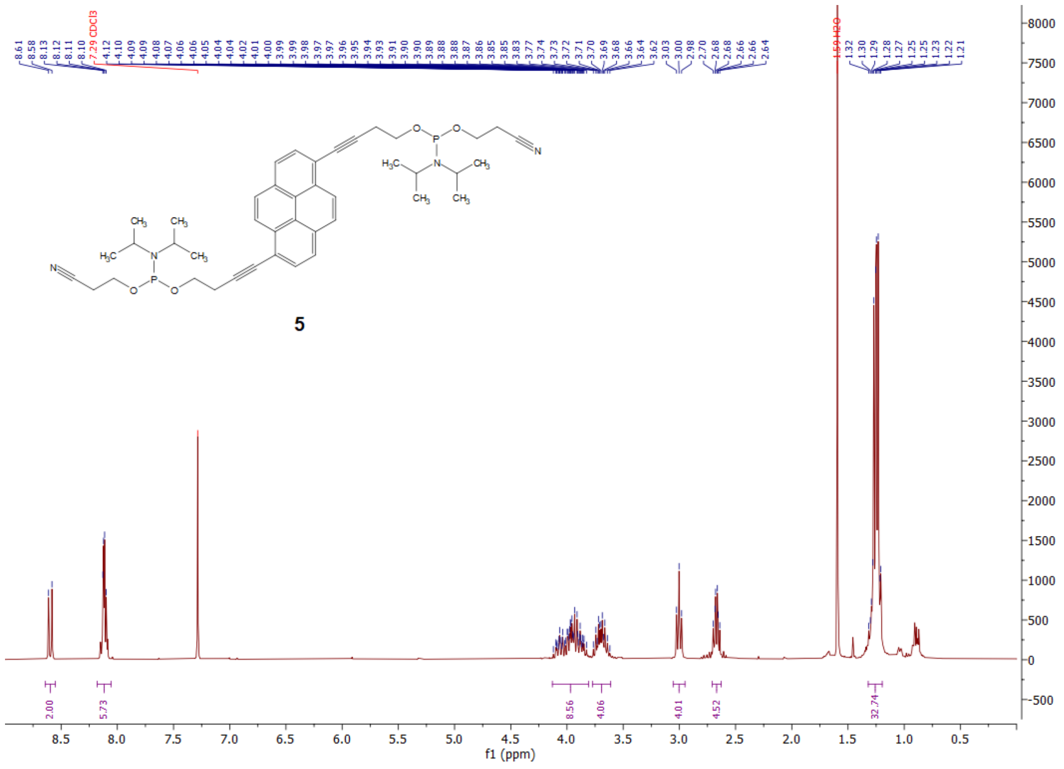

5

Figure S8 ^{1}H NMR spectra of **5**.

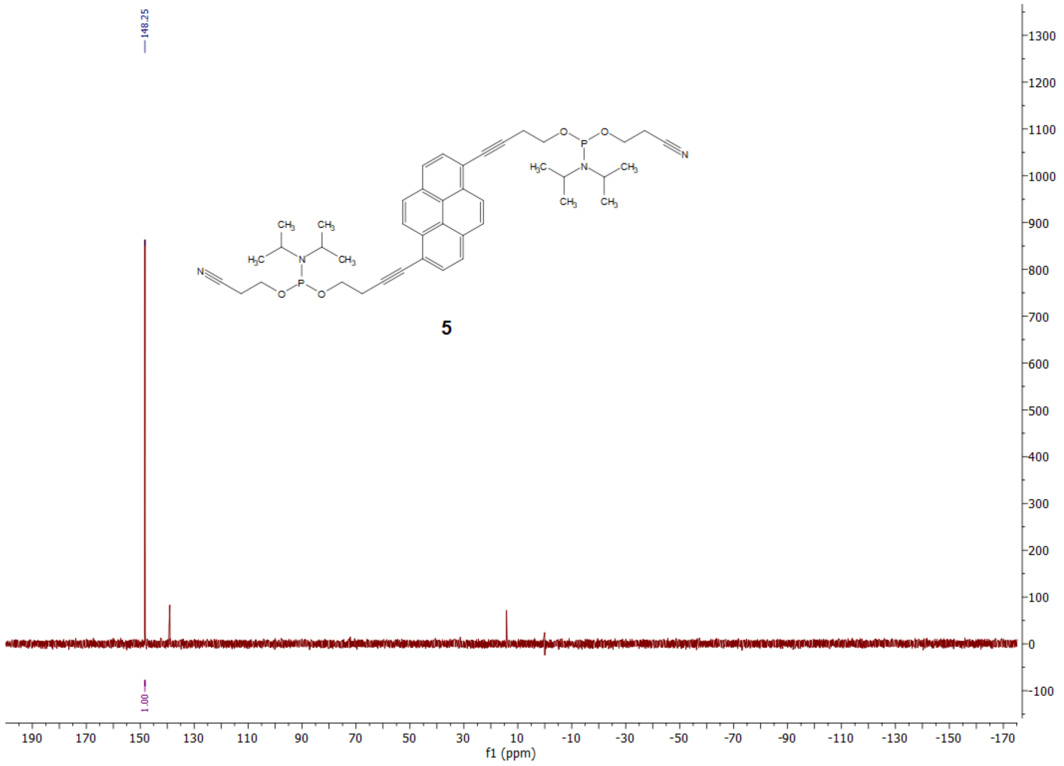

5

Figure S9 ^{31}P NMR spectra of **5.**

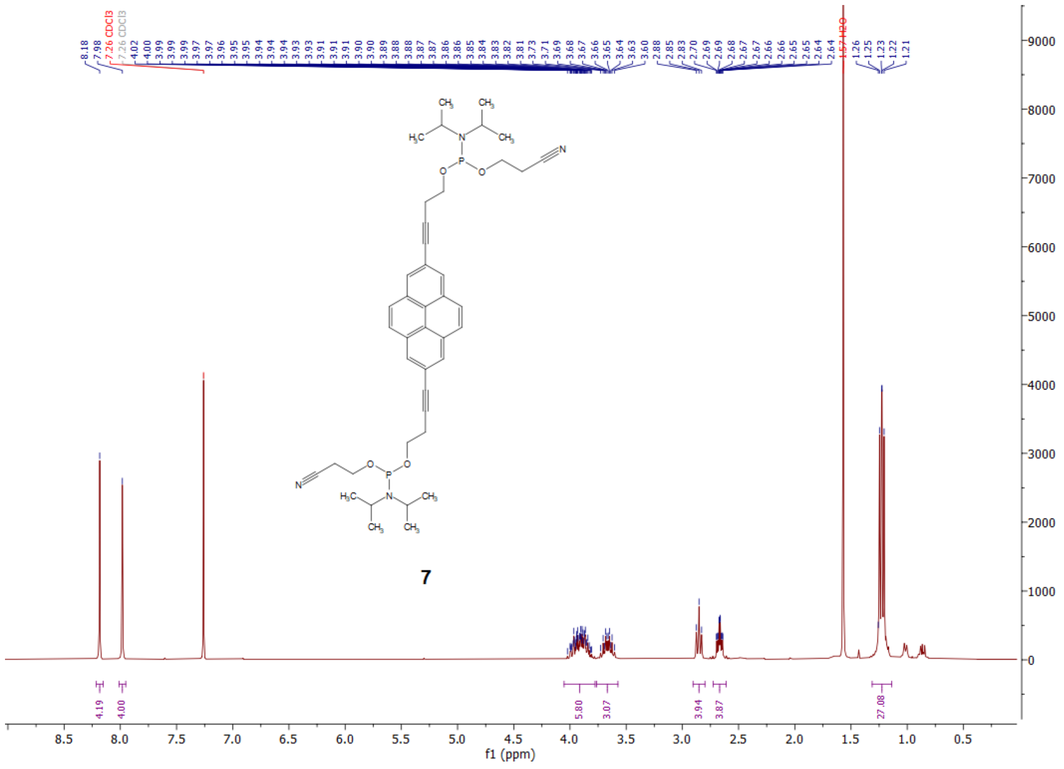

Figure S10 ^{1}H NMR spectra of **7.**

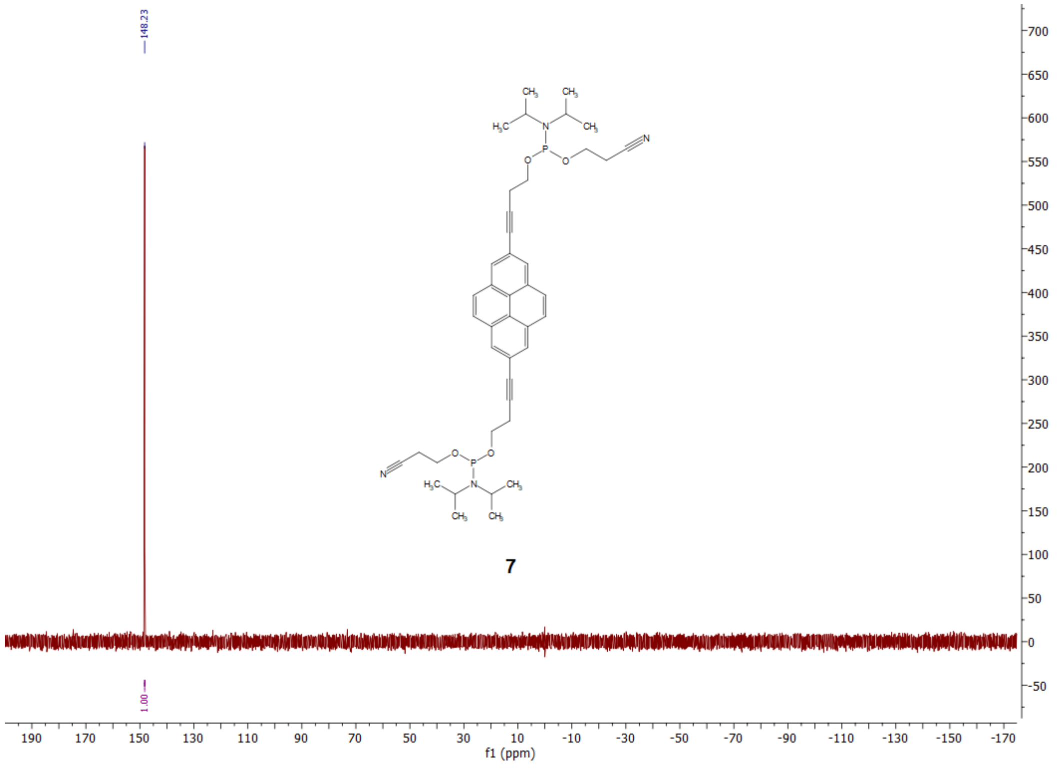

Figure S11 ^{31}P NMR spectra of **7**.

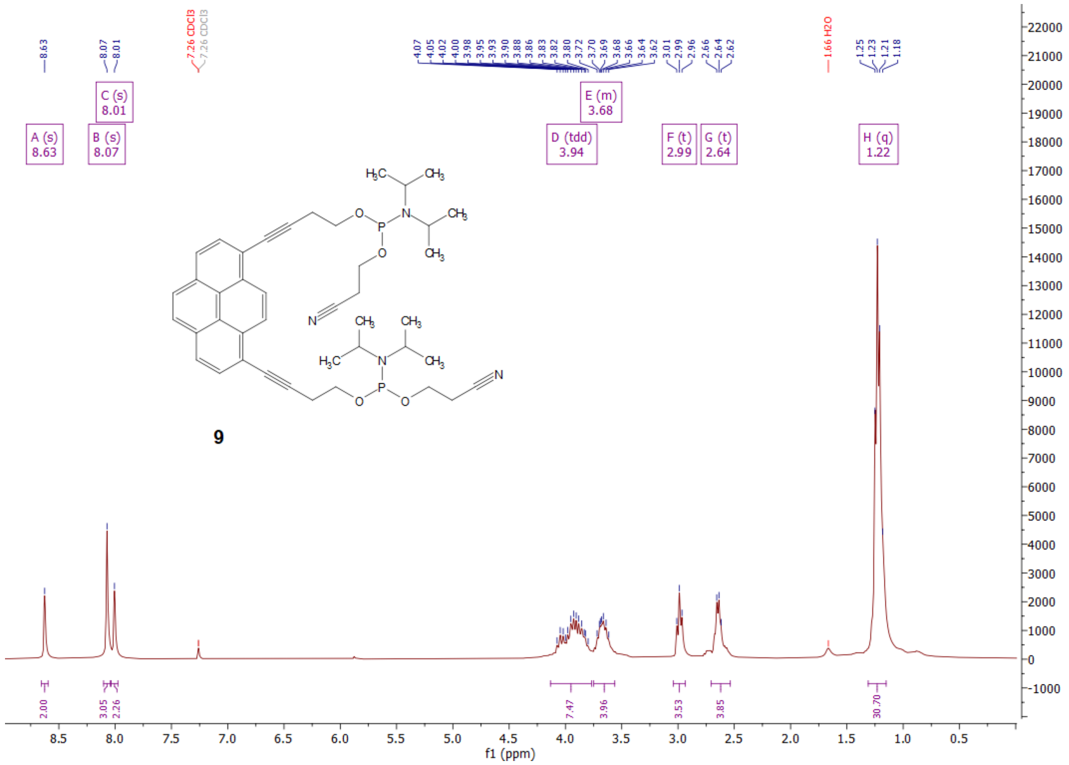

Figure S12 ^{1}H NMR spectra of **9.**

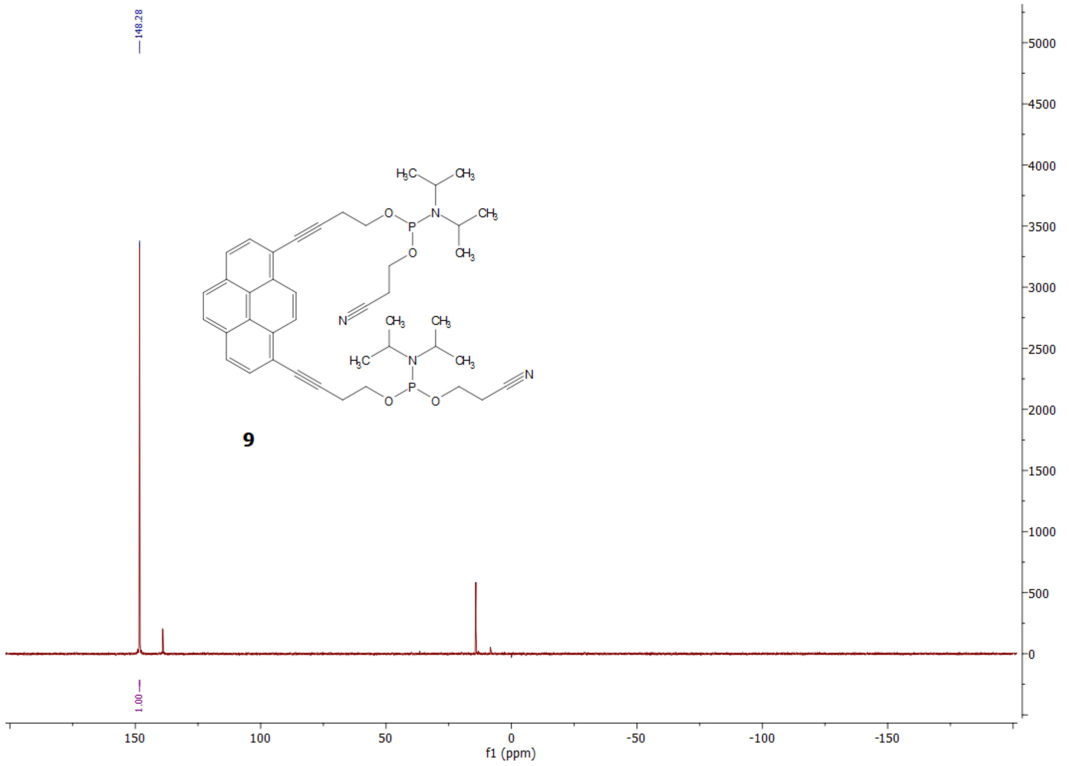

Figure S13 ^{31}P NMR spectra of **9**.

2.3. MS Spectra

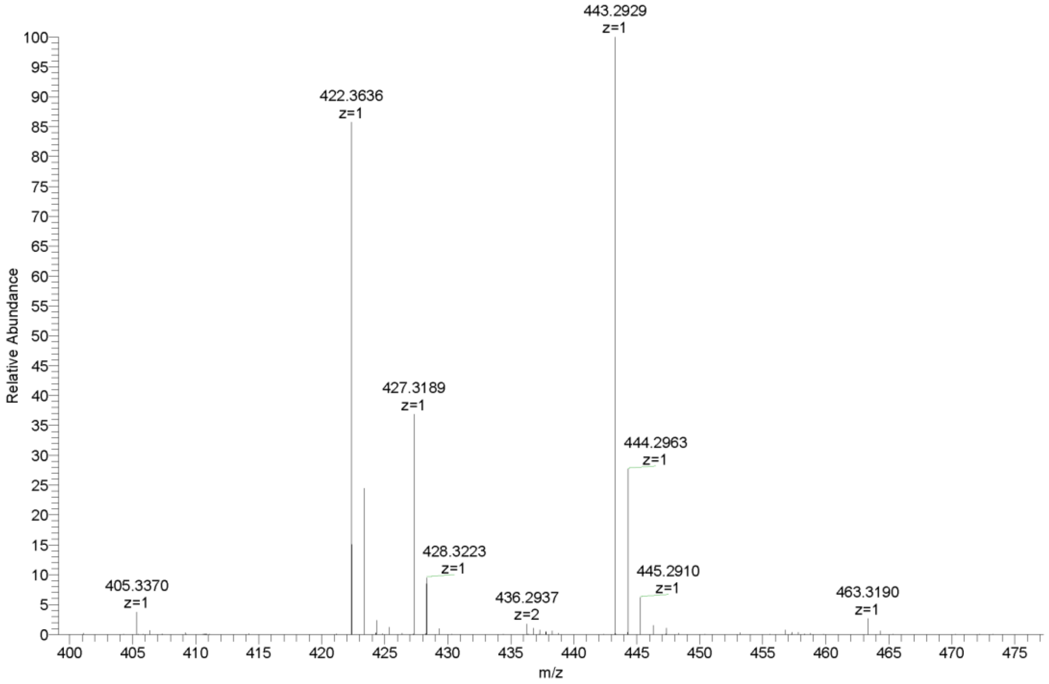

Figure S14 HR-MS for **3** in presence of a K^+ adduct.

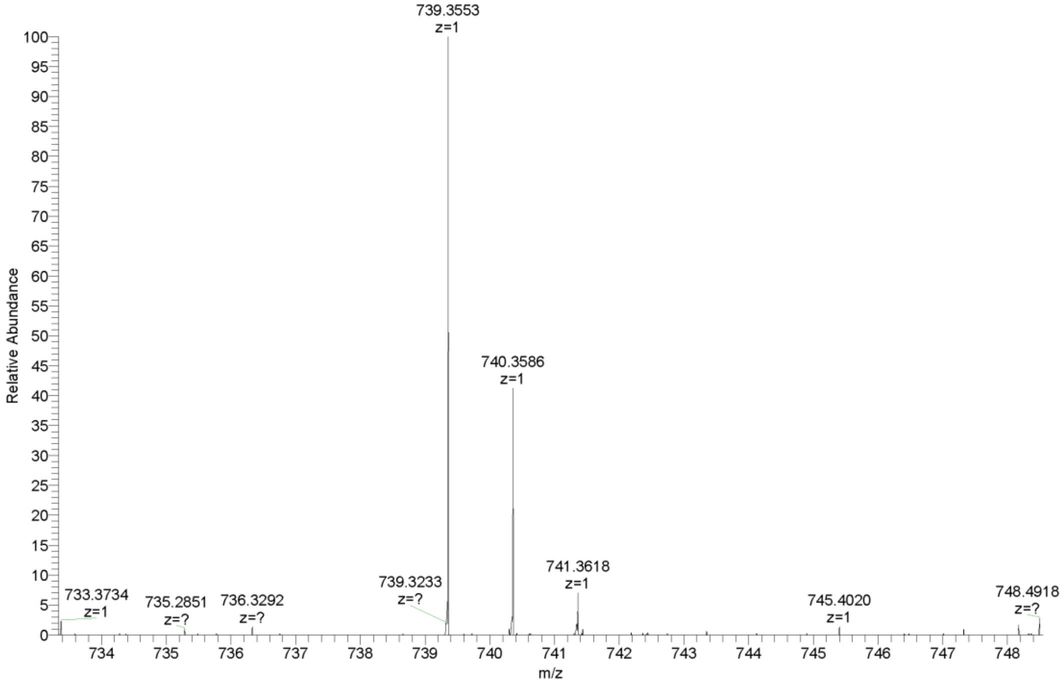

Figure S15 HR-MS of compound **5**.

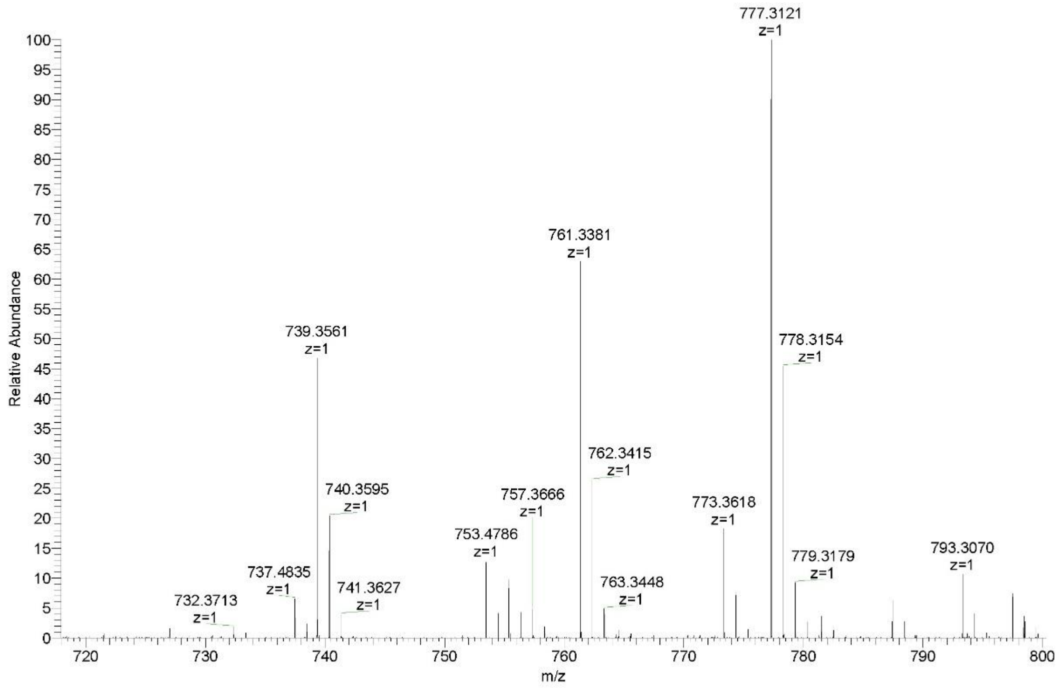

Figure S16 HR-MS for compound **7**.

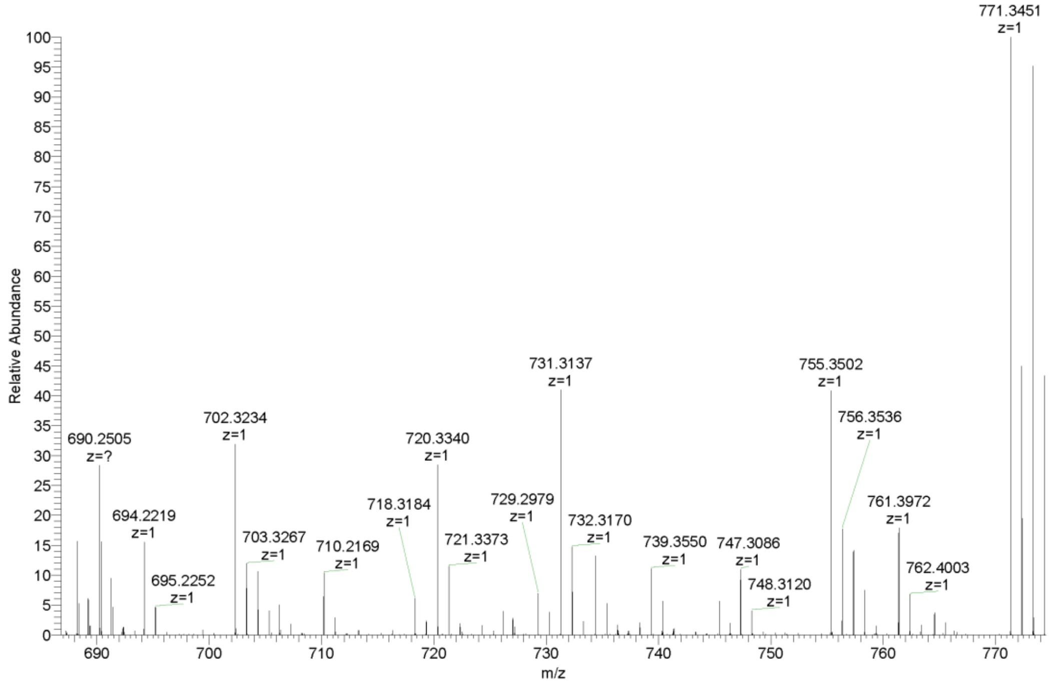

Figure S17 HR-MS of compound **9**.

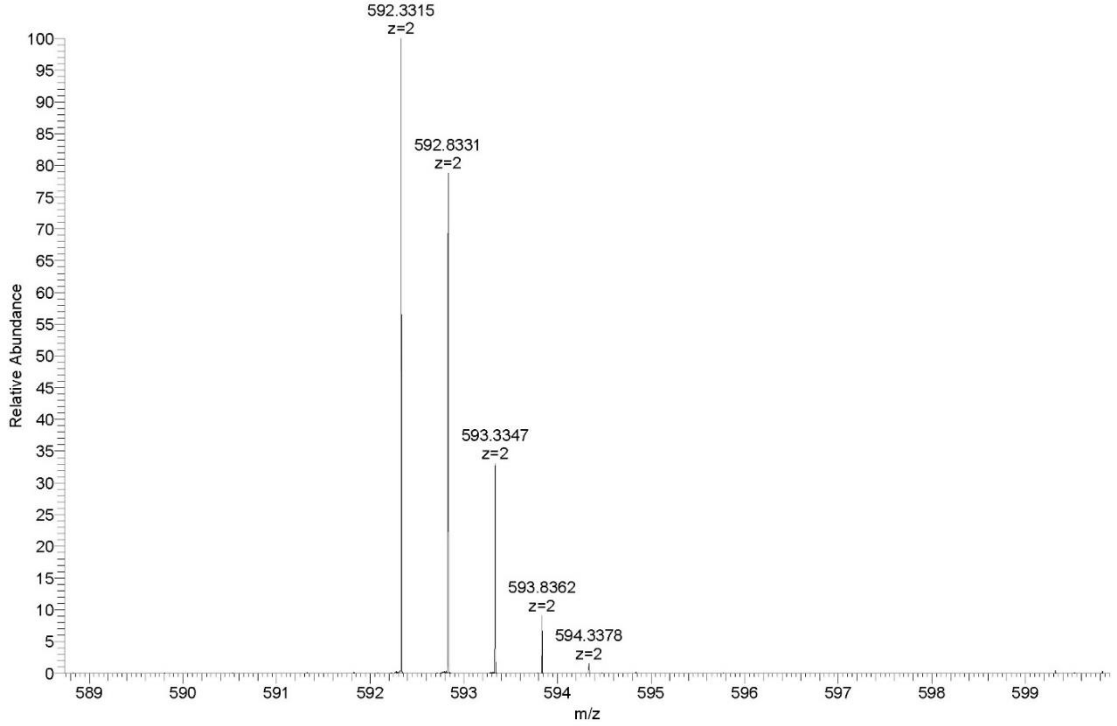

Figure S18 HR-MS of oligomer **CPC1**.

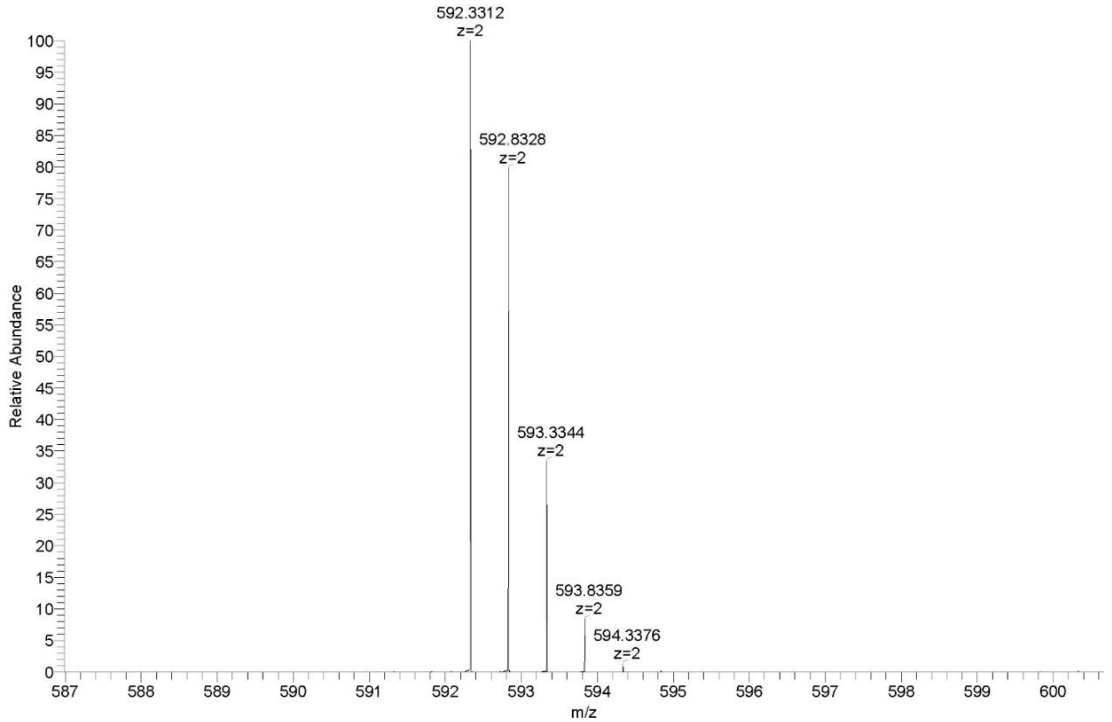

Figure S19 HR-MS of oligomer **CPC2**.

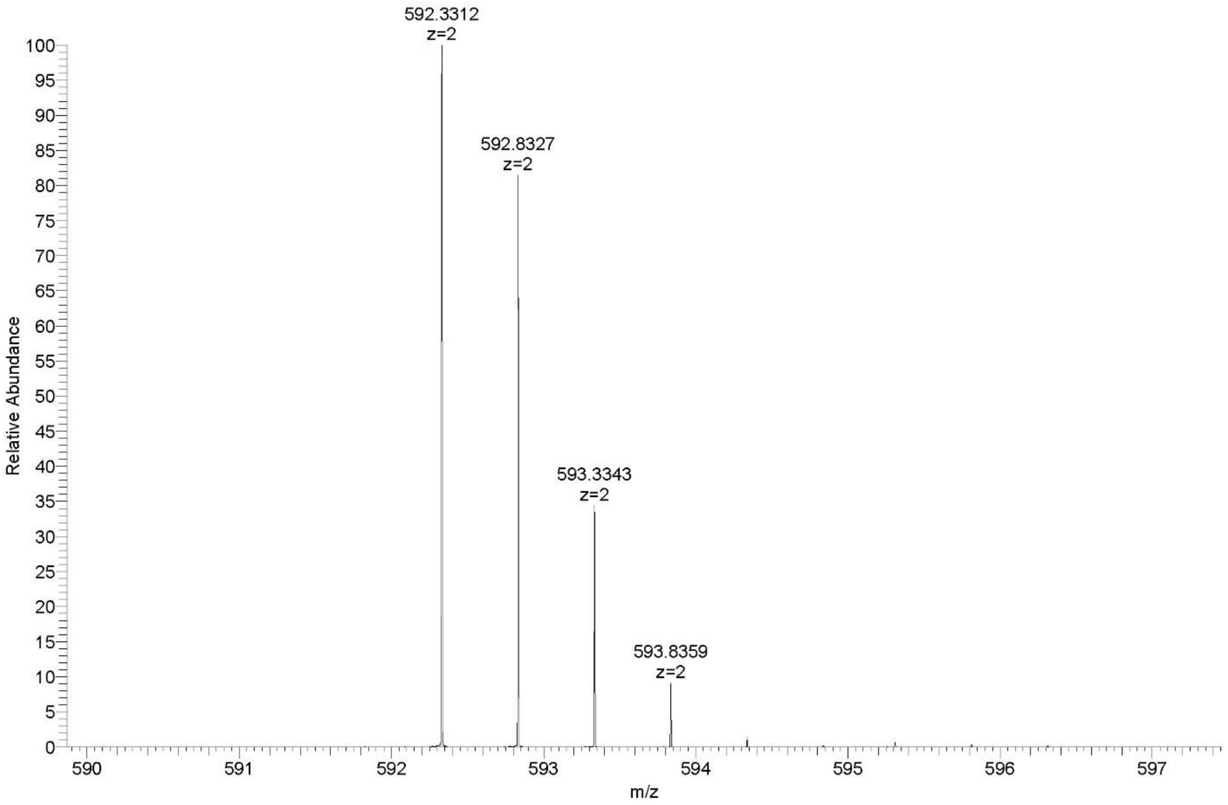

Figure S20 HR-MS of oligomer **CPC3**.

2.4. Analytical HPLC Trace

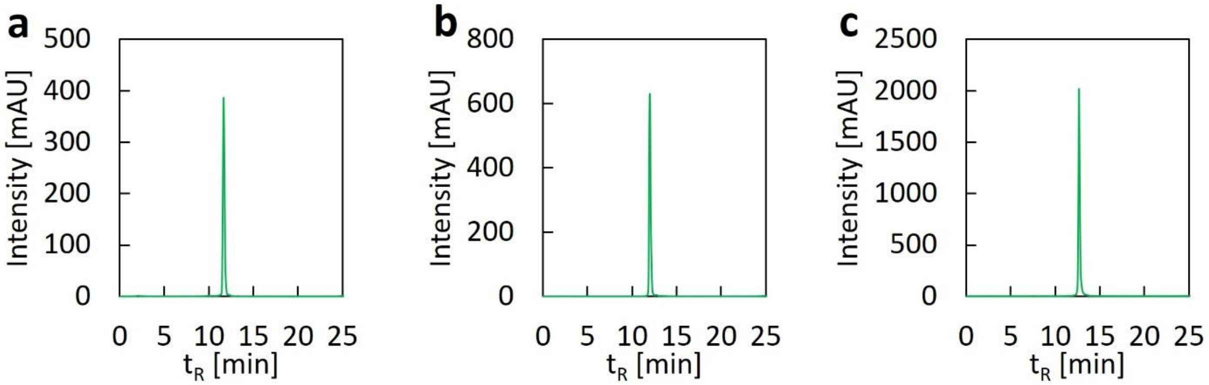

Figure S21 HPLC traces of (a) **CPC1**, $t_{[R]}$: 11.7 min ; (b) **CPC2**, $t_{[R]}$: 12.0 min ; (c) **CPC3**, $t_{[R]}$: 12.7 min.

3. Preparation of the sample

In an Eppendorf was added subsequently Milli-Q H_2O, EtOH, 100 µL of a sodium phosphate buffer (0.1 M, pH 7.2), 5 µL of NaCl (2 M) and finally the oligomer for a final volume of 1 mL. The solution was then vortexed to ensure a thorough mixing before being transferred to a quartz cuvette.

4. Temperature-dependent UV-vis Spectra

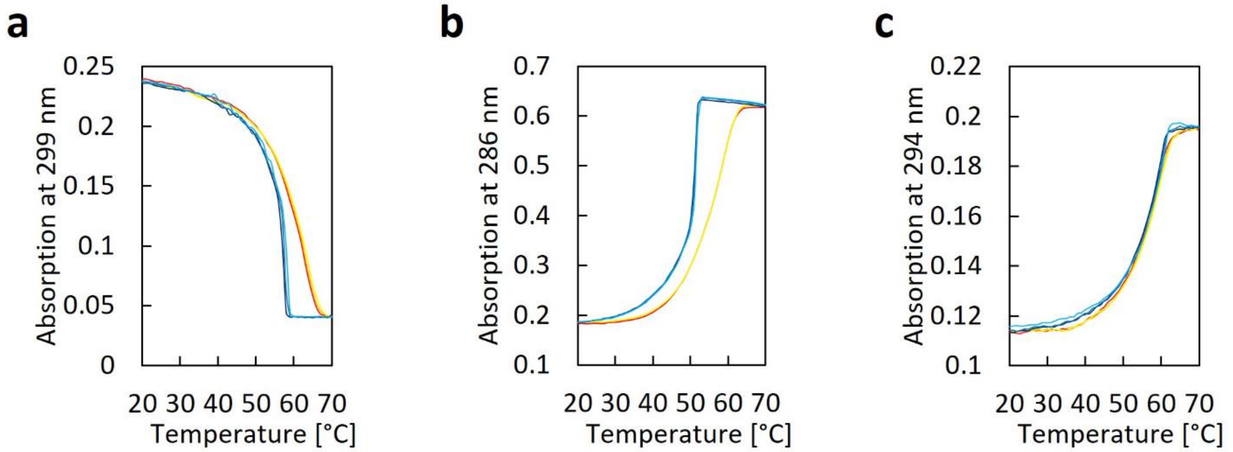

Figure S22 UV-Vis absorbance monitored at one specific wavelength upon cooling from 70 °C to 20 °C (blue colors) and heating to 70 °C (red and yellow colors) of (a) **CPC1** $\lambda_{abs.}$: 299 nm, (b) **CPC2** $\lambda_{abs.}$: 286 nm, (c) **CPC3** $\lambda_{abs.}$: 294 nm (gradient 0.5 °C·min^{-1}). Conditions: 3 µM oligomer, 10 mM sodium phosphate buffer, 10 mM NaCl and EtOH (20% for **CPC1**, 25% for **CPC2** and 15% for **CPC3**). A total of three consecutive cooling/heating cycles is shown with an interval of 5 minutes between each step.

5. Atomic Force Microscopy

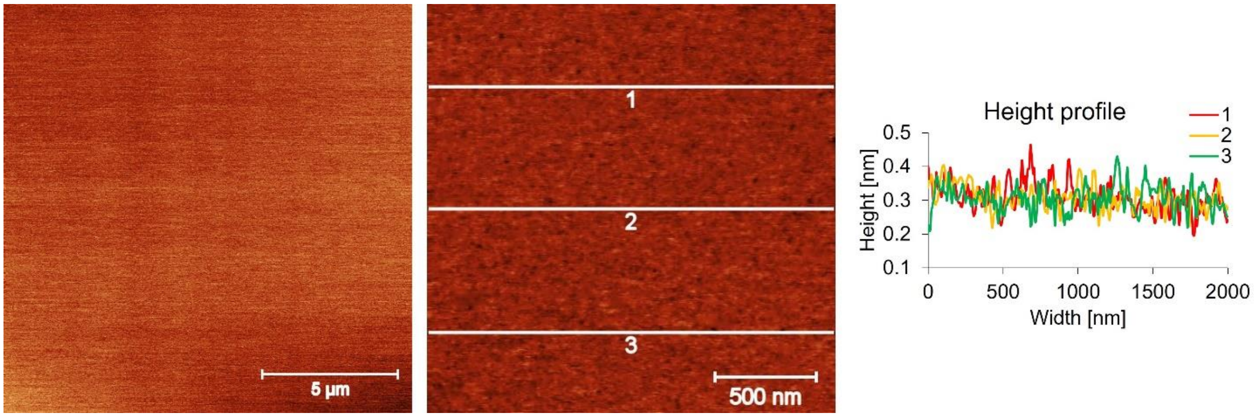

Figure S23 AFM images of a blank, APTES-modified mica sheet with cross section (right).

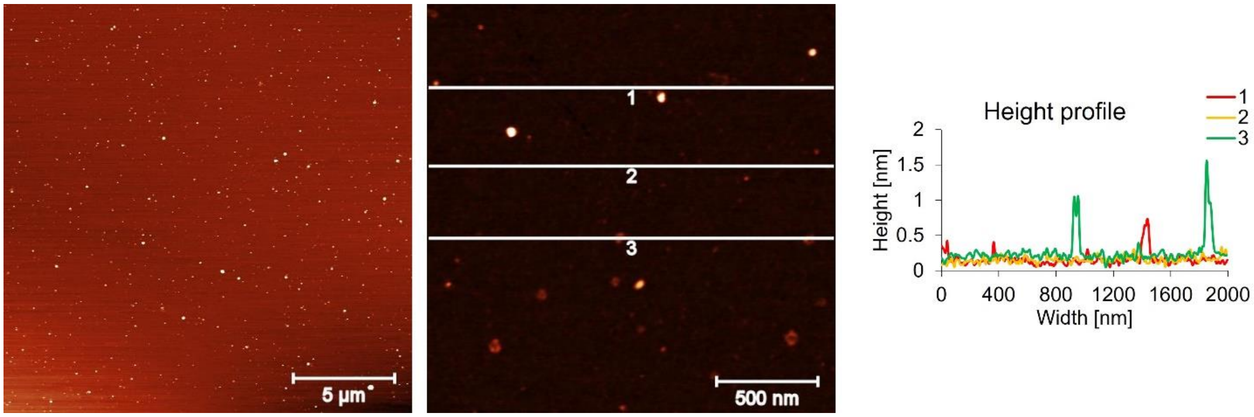

Figure S24 AFM images of the buffer used for **CPC1** on an APTES-modified mica sheet with cross section (right). Conditions: 3 µM oligomer, 10 mM sodium phosphate buffer, 10 mM NaCl and 20% EtOH.

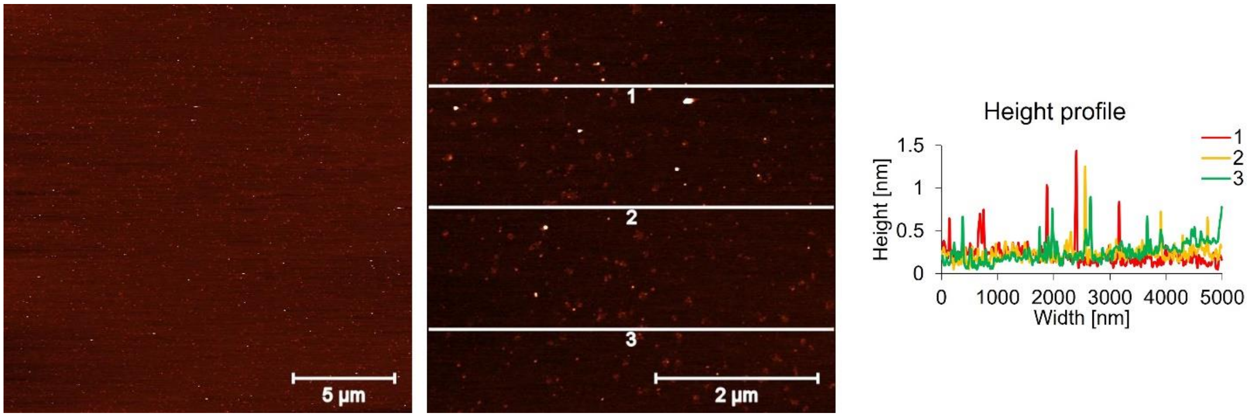

Figure S25 AFM images of the buffer used for **CPC2** on an APTES-modified mica sheet with cross section (right). Conditions: 3 µM oligomer, 10 mM sodium phosphate buffer, 10 mM NaCl and 25% EtOH.

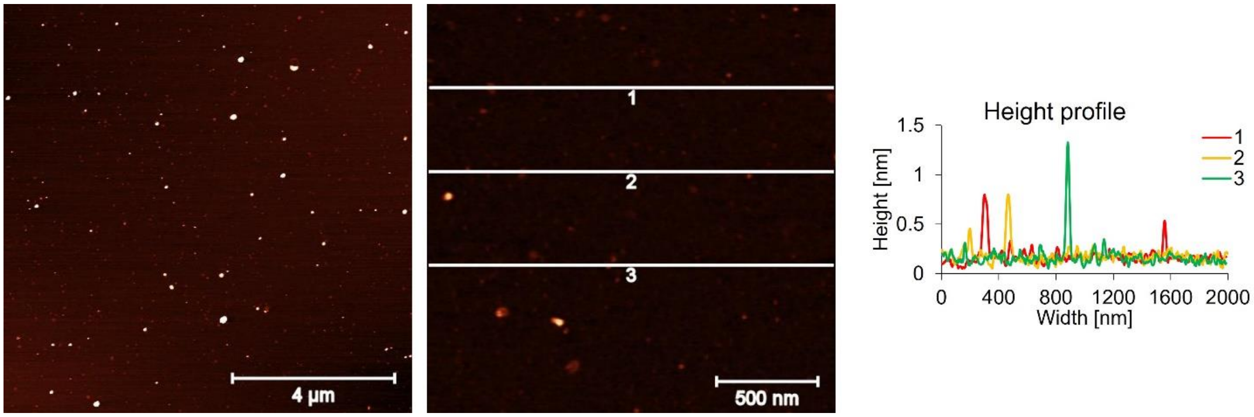

Figure S26 AFM images of the buffer used for **CPC3** on an APTES-modified mica sheet with cross section (right). Conditions: 3 µM oligomer, 10 mM sodium phosphate buffer, 10 mM NaCl and 15% EtOH.

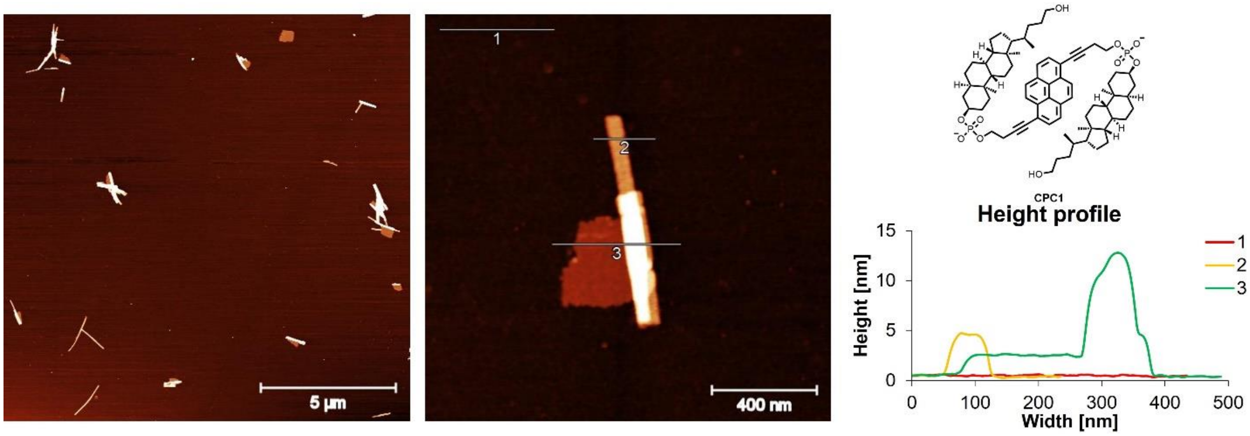

Figure S27 AFM images of the self-assembled **CPC1** on APTES-modified mica with cross section (right). Conditions: 3 µM oligomer, 10 mM sodium phosphate buffer, 10 mM NaCl and 20% EtOH.

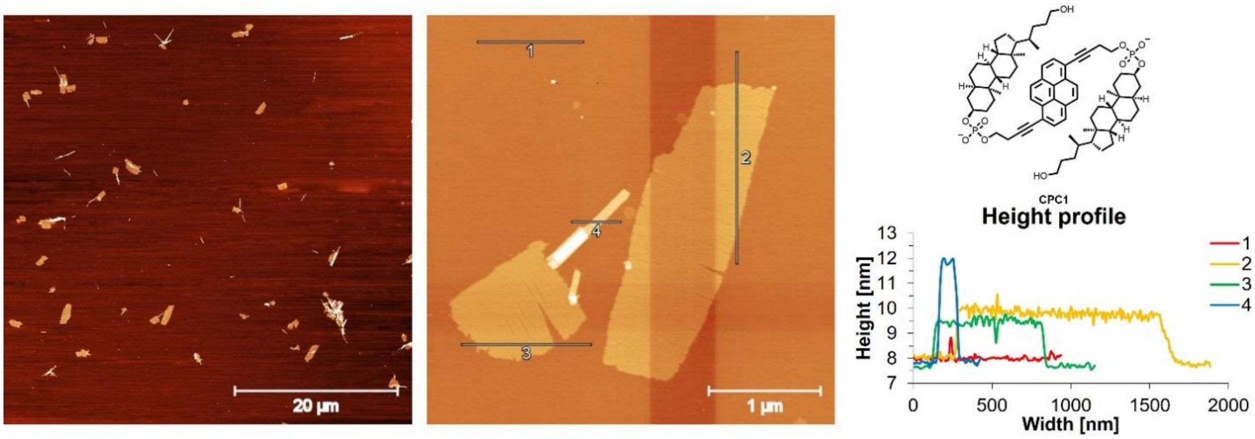

Figure S28 AFM images of the slow self-assembled **CPC1** (temperature gradient: 0.1°C per min) on APTES-modified mica with cross section (right). Conditions: 3 µM oligomer, 10 mM sodium phosphate buffer, 10 mM NaCl and 20% EtOH.

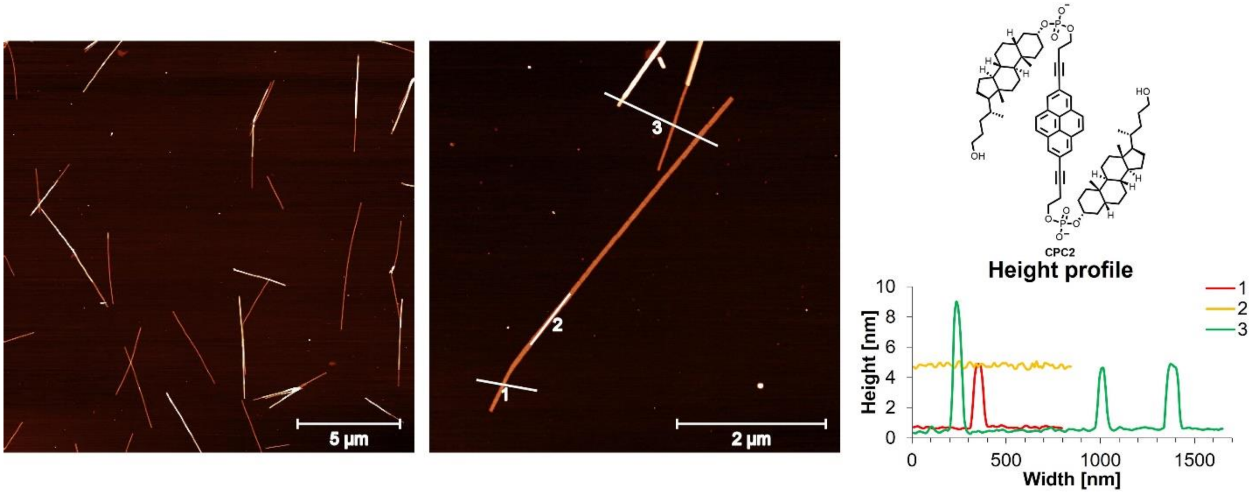

Figure S29 AFM images of the self-assembled **CPC2** on APTES-modified mica with cross section (right). Conditions: 3 µM oligomer, 10 mM sodium phosphate buffer, 10 mM NaCl and 25% EtOH.

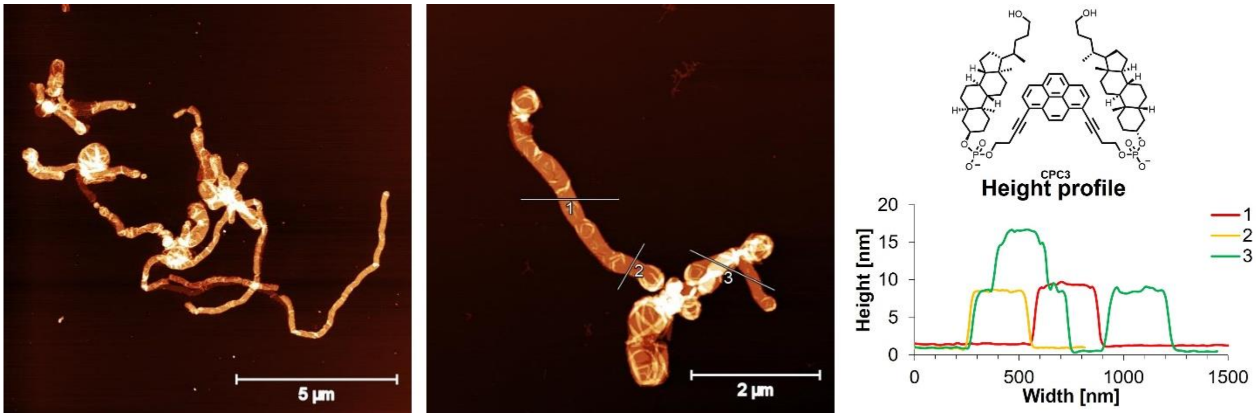

Figure S30 AFM images of the self-assembled **CPC3** on APTES-modified mica with cross section (right). Conditions: 3 µM oligomer, 10 mM sodium phosphate buffer, 10 mM NaCl and 15% EtOH.

6. Cryo-EM

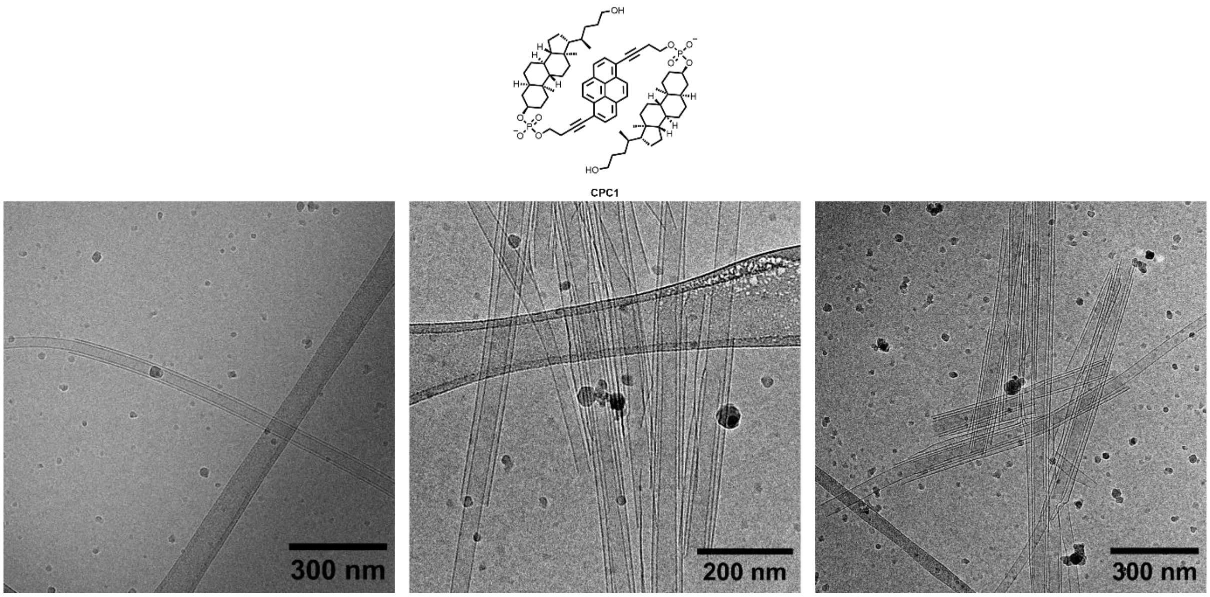

Figure S31 Cryo-EM images of **CPC1**. Conditions: 3 µM oligomer, 10 mM sodium phosphate buffer, 10 mM NaCl and 20% EtOH.

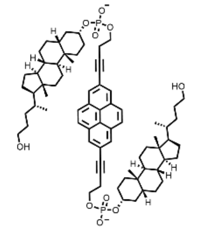

CPC2

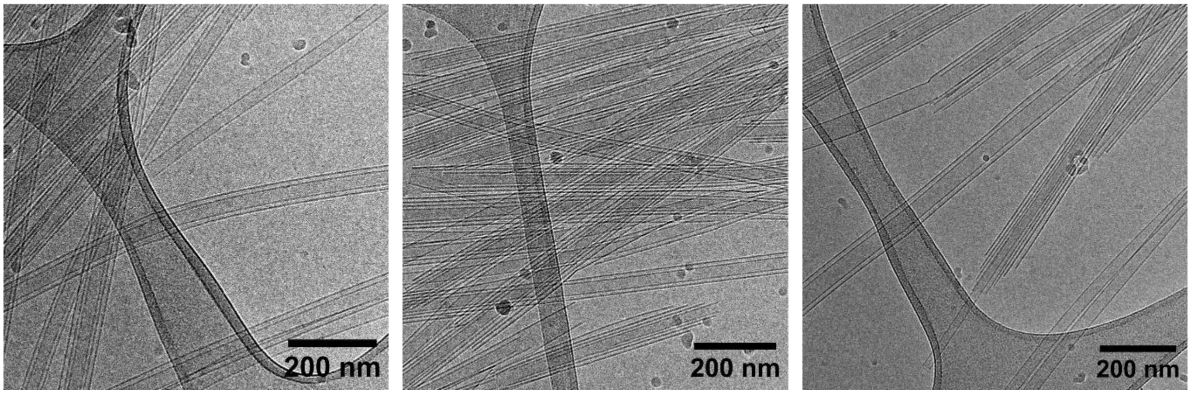

Figure S32 Cryo-EM images of **CPC2**. Conditions: 3 µM oligomer, 10 mM sodium phosphate buffer, 10 mM NaCl and 25% EtOH.

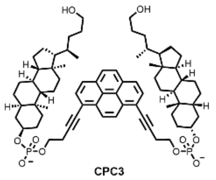

CPC3

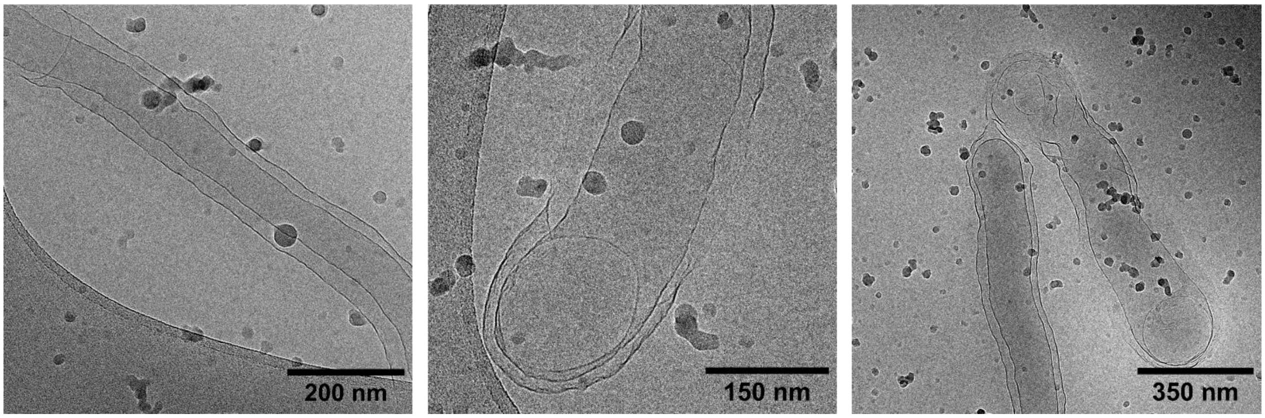

Figure S33 Cryo-EM images of **CPC3**. Conditions: 3 µM oligomer, 10 mM sodium phosphate buffer, 10 mM NaCl and 15% EtOH.

7. References

1 S. Rothenbühler, I. Iacovache, S. M. Langenegger, B. Zuber and R. Häner, *Nanoscale*, 2020, **12**, 21118–21123.

2 M. Linkert, C. T. Rueden, C. Allan, J.-M. Burel, W. Moore, A. Patterson, B. Loranger, J. Moore, C. Neves, D. MacDonald, A. Tarkowska, C. Sticco, E. Hill, M. Rossner, K. W. Eliceiri and J. R. Swedlow, *J. Cell Biol.*, 2010, **189**, 777–782.

3 J. Schindelin, I. Arganda-Carreras, E. Frise, V. Kaynig, M. Longair, T. Pietzsch, S. Preibisch, C. Rueden, S. Saalfeld, B. Schmid, J.-Y. Tinevez, D. J. White, V. Hartenstein, K. Eliceiri, P. Tomancak and A. Cardona, *Nat. Methods*, 2012, **9**, 676–682.

4 S. J. Kim, E.-K. Bang, H. J. Kwon, J. S. Shim and B. H. Kim, *ChemBioChem*, 2004, **5**, 1517–1522.
